# The *Toxoplasma* Centrocone Houses Cell Cycle Regulatory Factors

**DOI:** 10.1128/mBio.00579-17

**Published:** 2017-08-22

**Authors:** Anatoli Naumov, Stella Kratzer, Li-Min Ting, Kami Kim, Elena S. Suvorova, Michael W. White

**Affiliations:** a Department of Global Health and Florida Center for Drug Discovery and Innovation, University of South Florida, Tampa, Florida, USA; b Departments of Medicine, Pathology, and Microbiology & Immunology, Albert Einstein College of Medicine, Bronx, New York, USA; University of Wisconsin–Madison; Stanford University

**Keywords:** E3 ligase, *Toxoplasma gondii*, apicomplexan parasites, cell cycle, chromosome replication, cyclin-dependent kinases

## Abstract

Our knowledge of cell cycle regulatory mechanisms in apicomplexan parasites is very limited. In this study, we describe a novel *Toxoplasma gondii* factor that has a vital role in chromosome replication and the regulation of cytoplasmic and nuclear mitotic structures, and we named this factor ECR1 for essential for chromosome replication 1. ECR1 was discovered by complementation of a temperature-sensitive (ts) mutant that suffers lethal, uncontrolled chromosome replication at 40°C similar to a ts mutant carrying a defect in topoisomerase. ECR1 is a 52-kDa protein containing divergent RING and TRAF-Sina-like zinc binding domains that are dynamically expressed in the tachyzoite cell cycle. ECR1 first appears in the unique spindle compartment of the *Apicomplexa* (centrocone) of the nuclear envelope in early S phase and then in the nucleus in late S phase where it reaches maximum expression. Following nuclear division, but before daughter parasites separate from the mother parasite, ECR1 is downregulated and is absent in new daughter parasites. The proteomics of ECR1 identified interactions with the ubiquitin-mediated protein degradation machinery and the minichromosome maintenance complex, and the loss of ECR1 led to increased stability of a key member of this complex, MCM2. ECR1 also forms a stable complex with the cyclin-dependent kinase (CDK)-related kinase, *T*. *gondii* Crk5 (TgCrk5), which displays a similar cell cycle expression and localization during tachyzoite replication. Importantly, the localization of ECR1/TgCrk5 in the centrocone indicates that this *Apicomplexa*-specific spindle compartment houses important regulatory factors that control the parasite cell cycle.

## IMPORTANCE

Parasites of the apicomplexan family are important causes of human disease, including malaria, toxoplasmosis, and cryptosporidiosis. Parasite growth is the underlying cause of pathogenesis, yet despite this importance, the molecular basis for parasite replication is poorly understood. Filling this knowledge gap cannot be accomplished by mining recent whole-genome sequencing data because apicomplexan cell cycles differ substantially and lack many of the key regulatory factors of well-studied yeast and mammalian cell division models. We have utilized forward genetics to discover essential factors that regulate cell division in these parasites using the *Toxoplasma gondii* model. An example of this approach is described here with the discovery of a putative E3 ligase/protein kinase mechanism involved in regulating chromosome replication and mitotic processes of asexual stage parasites.

## INTRODUCTION

Highly efficient asexual replication is fundamental to the ability of apicomplexan parasites to spread infections in their hosts, and this is evident by the fact that the actions of the best drugs used to combat these infections all reduce or block parasite proliferation. Unfortunately, existing therapies, particularly against malaria (1), are under constant pressure from acquired parasite drug resistance, and new treatments are continually needed. The peculiar proliferative cell cycles of *Apicomplexa* parasites differ substantially from the hosts they inhabit, and it seems safe to predict that a better understanding of the molecular basis of parasite cell division could yield new drug targets. For most apicomplexan species, replication is characterized by a sequence of two chromosome replication cycles (2). A single G_1_ phase that is roughly proportional in length to the number of parasites produced precedes the first chromosome cycle (S/M_n_ nuclear cycle) whose biosynthetic focus is genome replication followed by a single chromosome cycle (S/M_n+1_ budding cycle) that produces infectious parasites assembled internally (endopolygeny) or adjacent to the plasmalemma (schizogony) of the mother parasite (2). How genome fidelity is preserved through variable rounds of apicomplexan chromosome replication is not understood, as many known regulators of cell cycle progression in yeast and multicellular eukaryotes are missing in these parasites (3). Further, the basic checkpoint mechanisms that regulate the cell cycle transitions in *Apicomplexa* replication are also poorly understood. It is generally not known what molecular mechanisms control G_1_-to-S-phase commitment, S-phase progression, or chromosome segregation or what regulatory factors enable apicomplexan parasites to suspend budding until the last round of chromosome replication.

Analysis of many *Apicomplexa* genomes shows that genome mining by itself will not yield the molecular basis of cell cycle control in these parasites, which will require bench discoveries. To this end, the *Toxoplasma gondii* tachyzoite has provided a valuable model to study cell cycle mechanisms. The *T. gondii* tachyzoite utilizes an abbreviated cell cycle (endodyogeny) to produce two progeny from each mother cell (4). The phases of the tachyzoite cell cycle are defined (5, 6) and feature G_1_, S, and overlapping mitotic and cytokinetic processes that can be now distinguished with specific protein markers (2). Key cell cycle transitions where checkpoint mechanisms likely operate have also been revealed by the development of synchrony methods (6) and the study of cell cycle temperature-sensitive (ts) mutants (2, 7, 8). At a minimum, *T. gondii* tachyzoites possess mechanisms that control G_1_ progression and commitment to chromosome replication and that may regulate a short G_2_ phase that is characterized by an unusual 1.8N genome content (3). The discovery of a bipartite centrosome with independent cores regulating karyokinesis or cytokinesis indicate additional points of cell cycle control (2). These previous studies have paved the way for understanding the regulatory basis of cell division in these parasites. In this study, we begin to fill in these details with the discovery of a unique protein complex composed of a dual zinc finger protein and protein kinase belonging to the cyclin-dependent family (cyclin-dependent kinase [CDK]-related kinases [Crks]) that is involved in regulating chromosome replication in the tachyzoite. This protein complex is initially housed in the unique spindle compartment of the *Apicomplexa* called the centrocone where it may regulate the connection between nuclear and cytoplasmic mitotic structures that must interact in order to coordinate the complex events associated with the tachyzoite mitosis.

## RESULTS

### *Toxoplasma* chromosome replication and segregation require a conserved topoisomerase and a novel dual zinc finger protein.

**FIG 1:**
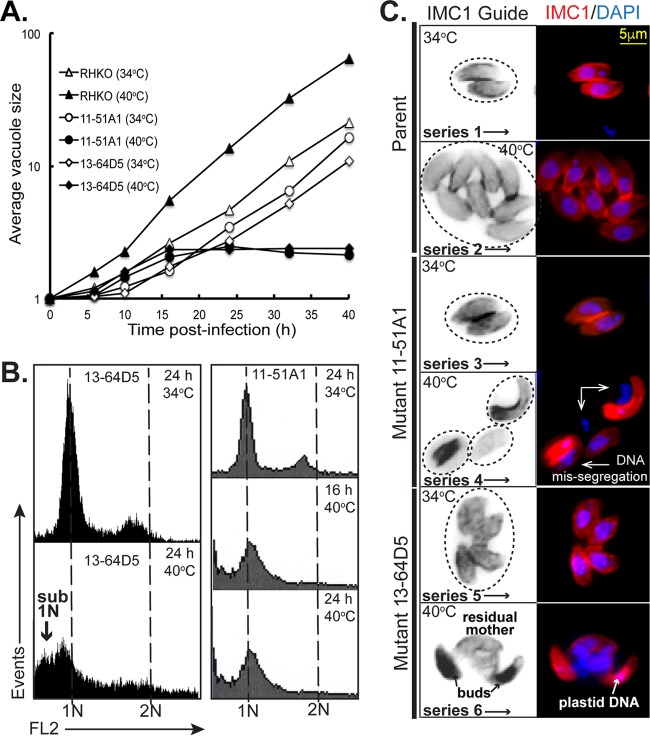
Temperature-sensitive (ts) mutants defective in chromosome replication. (A) The growth of chemical ts mutants 13-64D5 and 11-51A1 was inhibited by high temperatures leading to lethal arrest. At the nonpermissive temperature of 40°C, the ts mutants arrested within one or two divisions, whereas at the permissive temperature of 34°C, the ts mutants replicated similar to the parent strain. Note that all of the strains, including the parent strain, grew more slowly at 34°C compared to the parent strain at 40°C. The vacuole size shown on the *y* axis is the number of parasites per vacuole. RHKO, RHΔ*ku80*Δ*hxgprt* knockout strain. (B) Flow cytometric analysis of ethanol (EtOH)-fixed ts mutant parasites stained with propidium iodide after RNase treatment showed similar sub-1N aneuploidy that is consistent with chromosome loss at 40°C. The DNA content of the ts mutants grown at 34°C exhibits the 1N and 1.8N peaks representative of an asynchronous growing tachyzoite population (6). For each sample, 10,000 parasites were analyzed (*y* axis) using an FL-2 linear scale; 1N and 2N DNA fluorescent values are indicated on the *x* axis and by the vertical dashed lines. (C) IFA analysis of ts mutants 13-64D5 and 11-51A1 compared to the parent strain grown at 34°C (image series 1 to 5) and 40°C (image series 2 to 6). The parasites were stained for DNA with DAPI and internal daughter budding with anti-IMC1. The IMC1 Guide column show noncolored/inverted images in order to better differentiate individual mother parasites and daughter buds. The circles in the IMC1 Guide images indicate the number of vacuoles and the approximate size base of differential interference contrast (DIC) images (results not shown). Mutant images at 40°C (series 4 and 6) show a similar phenotype of unequal masses of chromosomal DNA accumulated free of abnormal daughter parasites. Note that the small DAPI focus in the buds of series 6 is plastid DNA. The scale bar in the series 1 fluorescent image applies to all images.

The peculiar cell cycles of the *Apicomplexa* have many unique features that suggest that molecular controls of replication in these parasites likely include novel mechanisms. To this end, we have used forward genetics to identify essential cell cycle factors in *T. gondii* (2, 7–11). The temperature-sensitive (ts) mutants 11-51A1 and 13-64D5 from our collection of ∼150 growth mutants (7) share similar lethal temperature defects (Fig. 1A, growth curves). When shifted to 40°C, both ts mutant parasites arrested quickly (within zero to two divisions), and this was associated with the loss of near-diploid-to-diploid genomic DNA content and the increase of daughter parasites possessing 1N and sub-1N contents as analyzed by flow cytometry (Fig. 1B). Cell division of each ts mutant strain at 34°C was similar to the parent (Fig. 1A and C), while at 40°C, ts mutants 11-51A1 and 13-64D5 showed severe defects in mitotic and cytokinetic processes (Fig. 1C) characterized by loss of normal nuclei and daughter parasite stoichiometry (normal ratios, 1:2 and 1:1). Daughter buds that often had plastid DNA formed, but many buds lacked nuclear DNA (Fig. 1C), indicating that cytokinesis and karyokinesis were uncoupled in both ts mutants. By immunofluorescence assay (IFA), masses of unpackaged nuclear DNA accumulated in the residual mother parasite (Fig. 1C), indicating that restrictions on chromosome replication may also be lost at high temperature. The accumulation of ts mutant parasites with sub-1N DNA contents observed in flow cytometry analysis (Fig. 1B) is consistent with the inability of these parasites to package their DNA into daughter parasites and the difficulty of recovering defective parasites that contain polyploid DNA masses.

**FIG 2:**
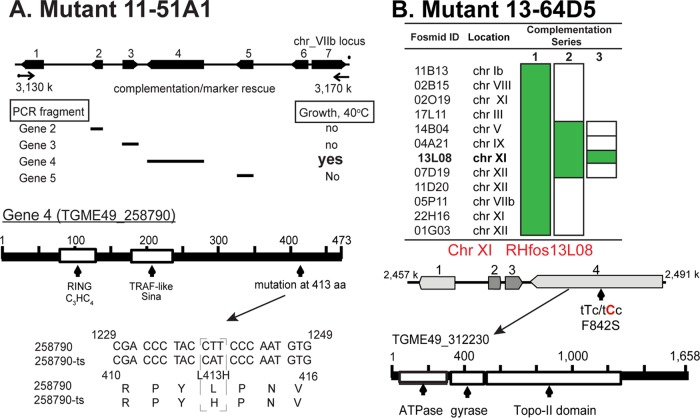
Identification of the defective genes in ts mutants 13-64D5 and 11-51A1. (A) Genetic complementation of ts mutant 11-51A1 with cosmid genomic libraries followed by marker rescue identified the defective locus to chromosome (chr) VIIb (see top diagram). The locus was further resolved using PCR products spanning each of the central four genes. Only gene 4 (TGME49_258790) rescued ts mutant 11-51A1 at 40°C and was sequenced to identify the ts mutation. The mutation and size and major protein domains for gene 4 are shown. aa, amino acid. (B) The genome of ts mutant 13-64D5 was sequenced to >150-fold coverage identifying 16 nonsynonymous mutations. Fosmid clones from a mapped *T. gondii* genetic library that spanned 12 of the sequenced mutations were combined and used to successfully complement ts mutant 13-64D5 (complementation series 1). Genetic complementation with fosmid clones remixed into three groups (series 2) followed by complementation of four individual fosmids (series 3) identified a region on chromosome XI as the locus carrying the mutation responsible for high temperature sensitivity. In the identified XI locus, ts mutant 13-64D5 harbors a single mutation (F842S) in a *T. gondii* ortholog of topoisomerase II (TgTopo-II) that is rescued by parental copy of TgTopo-II on fosmid RHfos13L08. Fosmid ID, fosmid identification.

To identify the defective gene in ts mutant 11-51A1, parasites were genetically complemented with a *T. gondii* cosmid-based genomic library under 40°C and pyrimethamine selection to ensure that rescued parasites possessed cosmid DNA (7). Marker rescue of the resulting transgenic parasites (without cloning) identified a locus on chromosome VIIb as the location of the ts mutation (Fig. 2A). Complementation of the original ts mutant 11-51A1 with PCR fragments spanning each of the four central genes in the locus determined that gene 4 (TGME49_258790) was the defective gene. Sequencing of ts mutant gene 4 pinpointed a nonsynonymous mutation that changed a leucine to histidine at residue 413 as the cause of temperature sensitivity in this chemical ts mutant. Gene 4 encodes an ∼52-kDa protein containing divergent RING (really interesting new gene) (Pfam accession no. PF13923; e−08) and TRAF-like Sina (TRAF stands for tumor necrosis factor receptor-associated factor, and Sina stands for seven *in absentia*) (e−09) zinc binding domains (Fig. 2A). Due to the requirement of this protein for proper tachyzoite chromosome replication and segregation, gene 4 was named ECR1 for essential for chromosome replication 1. Western analysis of ectopically expressed ECR1^Myc^ (ECR1 with Myc epitope tag) and temperature-sensitive ECR1^Myc^ (tsECR1^Myc^) revealed that protein instability at high temperature (see Fig. S1A in the supplemental material) was likely responsible for the loss of tsECR1 function and the underlying cause of temperature lethality of the original 11-51A1 ts mutant strain.

10.1128/mBio.00579-17.1 FIG S1  Regeneration of the tsECR1 phenotype by genetic knock-in. (A) Western analysis demonstrated that tsECR1^Myc^ is unstable at high temperature compared to ECR1^Myc^. (B) Strategy for introduction of the tsECR1 mutation (L413H) by genetic knock-in while simultaneously fusing a 3×Myc epitope tag to the C terminus. (C) IFA analysis of the recreated tsECR1^Myc^ ts mutant showed identical temperature-dependent growth characteristics manifested by severe chromosome defects at high temperature as observed in the original ts mutant 11-51A1. Download FIG S1, TIF file, 1.2 MB. Copyright © 2017 Naumov et al. 2017 Naumov et al. This content is distributed under the terms of the Creative Commons Attribution 4.0 International license.

In order to identify the defective gene in ts mutant 13-64D5, we used a whole-genome sequencing/complementation approach as previously described (12). Briefly, ts mutant 13-64D5 genomic DNA was sequenced to >150-fold coverage on the Illumina HiSeq 2500 platform, and nonsynonymous mutations were identified in 16 genes. Three of these genes were eliminated due to the lack of tachyzoite expression and/or a conserved amino acid substitution. Fosmid clone DNA carrying *T. gondii* genomic inserts (12) encompassing the remaining 13 genes (fosmid 02O19 spanned two single nucleotide polymorphisms [SNPs]) were purified and combined into a single mix for genetic complementation (Fig. 2B, complementation series 1). Successful rescue of ts mutant 13-64D5 with the combined 12 fosmid DNAs was followed by complementation with the 12 fosmid DNAs remixed in 3 groups of 4 (Fig. 2, complementation series 2), and finally, 4 individual fosmid clones were tested that identified a single fosmid clone able to rescue high temperature sensitivity (series 3). The sole ts mutant SNP in the genomic locus spanned by the fosmid clone RHfos13L08 insert is a TTC-to-TCC change that leads to serine substituting for phenylalanine at amino acid residue 842 in the *T. gondii* ortholog of eukaryotic topoisomerase II (TGME49_312230). *T. gondii* topoisomerase II (TgTopo-II) is a 185-kDa protein with N-terminal ATPase and gyrase domains, central topoisomerase domain, and unique C-terminal tail (Fig. 2B).

### TgTopo-II and ECR1 are cell cycle regulated nuclear factors.

TgTopo-II and ECR1 proteins represent distinct evolutionary histories in apicomplexan parasites (Fig. S2). Topo-II is highly conserved in eukaryotes and is present in all apicomplexan species sequenced (Fig. S2A). In contrast, ECR1 is limited to modern coccidian species (Fig. S2B). However, we found highly divergent ECR1 orthologs in ancestral free-living protozoan *Chromera velia* and, unexpectedly, in filamentous fungi (Fig. S2A), indicating that the last common ancestor of eukaryotes might have encoded ECR1-related factor. Marginal sequence similarity covers only functional regions of these hypothetical proteins, namely, a RING and a TRAF-like Sina domain (Fig. S2B). Eukaryotic Sina factors have been proposed to link DNA damage to β-catenin degradation, providing a mechanism that senses altered DNA in cells (13). ECR1 may be related to these factors.

10.1128/mBio.00579-17.2 FIG S2  Phylogenetic analysis of *T*. *gondii* topoisomerase II and ECR1. (A) Conservation of the *T. gondii* topoisomerase II was confirmed by phylogenetic analysis that included representative apicomplexan species and several model eukaryotes. (B) Molecular phylogeny of *T. gondii* ECR1 protein compared to other coccidian species of apicomplexans, filamentous fungus (*Phycomyces blakesleeanus*) and ancestral alveolata (*Chromera velia*) is shown. Since ECR1 orthologs were not found in higher eukaryotes, human PDZRN3 factor that similar to ECR1 contains N-terminal RING and TRAF-like Sina domains was used as an outgroup. The span of the region of homology between ECR1 and the fungal counterpart is shown on the top schematics. Red numbers represent support values for the node obtained from 100 bootstrap replicates. The trees are drawn to scale, with branch lengths determined by the number of substitutions per site also shown in the scale. Download FIG S2, TIF file, 1.3 MB. Copyright © 2017 Naumov et al. 2017 Naumov et al. This content is distributed under the terms of the Creative Commons Attribution 4.0 International license.

**FIG 3:**
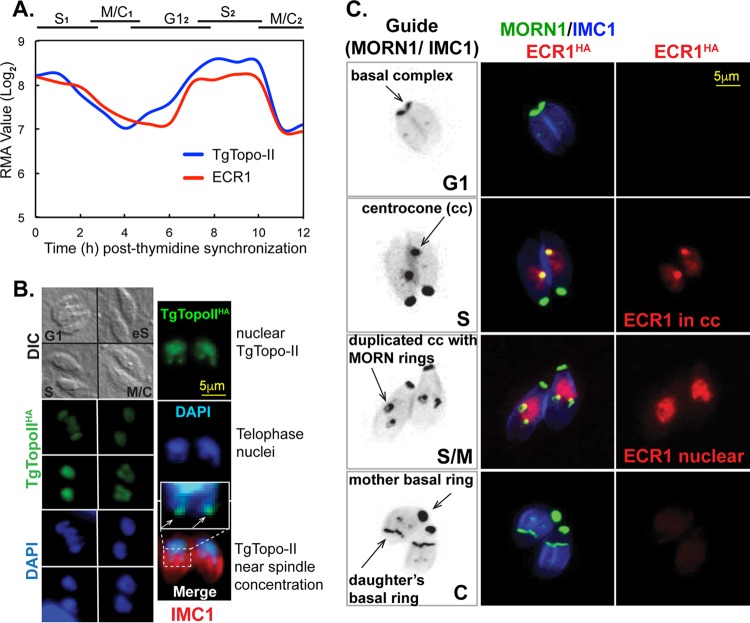
ECR1 and TgTopo-II are cell cycle regulated nuclear factors. (A) The mRNAs encoding ECR1 and TgTopo-II are similarly regulated by the cell cycle, with peak expression in S phase consistent with potential roles in DNA replication (all mRNA data from our cell cycle transcriptome and deposited and presented by ToxoDB) (21). RMA values, robust multiarray average values for mRNA levels. (B and C) The wild-type genes ECR1 (defective in ts mutant 11-51A1) and TgTopo-II (defective in mutant 13-64D5) were epitope tagged with 3×HA by genetic knock-in. IFA an alysis of TgTopo-II^HA^ and ECR1^HA^ revealed a cell cycle expression profile matching their respective cyclical mRNA profiles. Both factors were maximally expressed during S phase before falling to low or undetected levels in early G_1_ parasites. (B) TgTopo-II^HA^ transgenic parasites were costained with anti-HA (green), anti-IMC1 (red), and DAPI (blue) to visualize DNA. The DIC image at the top left shows four vacuoles in different cell cycle phases as indicated (eS, early stationary). TgTopo-II^HA^ was exclusively nuclear throughout the cell cycle with some protein concentrating in the leading edge of nuclei (inset in the merged panel) beginning to undergo nuclear division (telophase). (C) ECR1^HA^ (red) transgenic parasites were costained for MORN1 (green) to visualize the centrocone (cc) and basal complexes and also for IMC1 to reveal the mother and daughter inner membrane complexes (blue). The cell cycle expression of ECR1^HA^ was complex; first appearance in the centrocone in early S, then exclusively nuclear in late S, and disappearing prior to nuclear division. Guide panels are noncolorized/inverted IMC1/MORN1 merged images labeled for centrocone (spindle pole) and basal complexes (front edge of growing daughter buds). The cell cycle phases are indicated in the lower right corners of the guide panels. The indicated scale bars apply to all images in each series in panels B and C.

**FIG 4:**
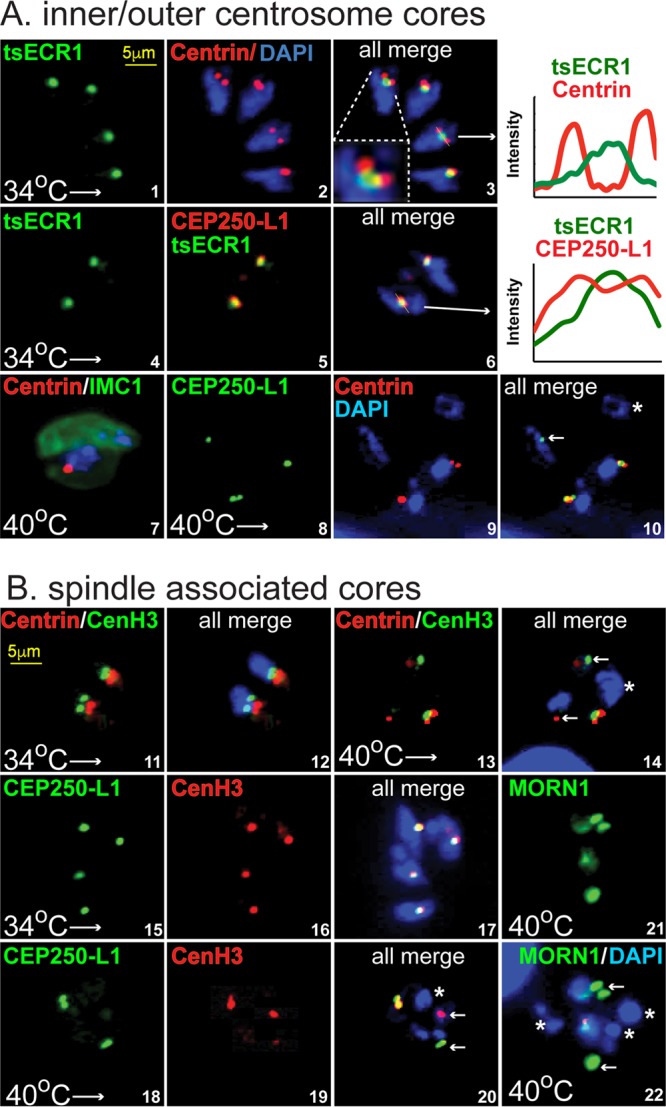
ECR1 is required for replicating and assembling key mitotic structures. Mitotic structures were examined in tsECR1^HA^ ts mutant parasites (see Fig. S1B for construction) at permissive and nonpermissive temperatures. Visualization of the inner (CEP250-L1^HA^) and outer (anticentrin) cores of the tachyzoite centrosome (2) and the centrocone (tsECR1^HA^ at 34°C) and kinetochore (anti-CenH3) was accomplished by epitope tagging specific integral proteins (by genetic knock-in) or by staining with protein-specific antibodies as indicated. DAPI staining was used to visualize genomic DNA content, and scale bars are indicated once in each series. (A) Centrosome structures (centrosome inner and outer cores) of growing tsECR1^Myc^ mutant parasites at 34°C (images 1 to 6) were compared to growth-arrested parasites at 40°C (images 7 to 10). The following pairs of proteins were evaluated at 34°C: images 1 to 3, tsECR1^Myc^ in the centrocone (green) versus centrin expression (red) in the outer centrosome core; images 4 to 6, tsECR1^Myc^ (green) in the centrocone versus CEP250-L1^HA^ in the centrosome inner core (each red stains). The inset in image 3 and the graphs of fluorescent scans (lines through the structures indicated by arrows) in images 3 and 6 show tsECR^Myc^ localization in relation to centrin in the outer and CEP250-L1^HA^ in the inner centrosome cores. Note the clear physical resolution of tsECR1^Myc^ in the centrocone from centrin in the centrosome outer core. The following pairs of protein expressed at 40°C were evaluated: image 7, centrin versus IMC1 in the tsECR1^Myc^ mutant strain (tsECR1^Myc^ is absent in these parasites at 40°C); images 8 to 10, CEP250-L1^HA^ versus centrin expression in the tsECR1^Myc^ transgenic strain. Note the abnormal nuclei/core stoichiometry and loss of alignment of the centrosome cores. In image 10, the nucleus indicated by a white asterisk has no centrosome cores, while the nucleus indicated by a white arrow has only an inner core containing CEP250-L1^HA^. (B) Spindle-associated structures (centrocone, inner core, and kinetochore) of tsECR1^Myc^ mutant parasites at 34°C (images 11, 12, and 15 to 17) were compared to growth at 40°C (images 13, 14, and 18 to 22). Histone CenH3 of the kinetochore (green) was compared to centrin (red) of the outer centrosome core (images 11 to 14). Normal replication and inside/outside close alignment of these structures to the nucleus (DAPI staining [blue]) were observed in tsECR1^Myc^ parasites grown at 34°C (images 11 and 12), whereas the loss of tsECR1^Myc^ at 40°C lead to severe physical and stoichiometric changes. The white asterisk in image 14 marks a nucleus lacking both centrosome and kinetochore structures, and the white arrows indicate single, free kinetochore and outer core structures in the ts mutant parasite cytoplasm. The inner core protein CEP250-L1^HA^ was compared to CenH3 in parasites grown at 34°C (images 15 to 17) versus 40°C (images 18 to 20). Note that the normal tight alignment of the inner centrosome core with the kinetochore is lost in tsECR1^Myc^ parasites grown at high temperatures. In image 20, the white asterisk marks a nucleus lacking inner and kinetochore structures, while the white arrows mark single core structures of each type. In images 21 and 22, MORN1 versus DAPI staining in tsECR1^Myc^ parasites at 40°C possess nuclei (white asterisks) without an intact centrocone.

TgTopo-II and ECR1 mRNAs are similarly cell cycle regulated with peak expression in the tachyzoite S phase (Fig. 3A). To determine whether the profile of the encoded proteins was also periodic, each protein was C-terminally fused to three copies of the hemagglutinin (HA) epitope by genetic knock-in in order to preserve native expression. TgTopo-II^HA^ (TgTopo-II with HA epitope tag) was exclusively nuclear and expressed throughout the tachyzoite cell cycle with an increase in expression during S phase (Fig. 3B) similar to the encoded mRNA (Fig. 3A). TgTopo-II^HA^ colocalized with nuclear DNA (Fig. 3B, see DAPI [4′,6′-diamidino-2-phenylindole] and anti-HA costaining), and during telophase, this factor was concentrated at the tips of u-shaped nuclei beginning to divide (Fig. 3B, inset). Like TgTopo-II^HA^, ECR1 tagged by genetic knock-in was determined to be a nuclear factor, although the expression of ECR1^HA^ (ECR1 with HA epitope tag) was dynamically cell cycle regulated. ECR1^HA^ first appeared following duplication of the centrosome outer core (see for example Fig. 4, images 1 to 3) concentrated in the apicomplexan-specific centrocone prior to the duplication of this structure (Fig. 3C, see guide reference and MORN1 costain) (2). Following duplication and separation of the centrocone (early mitosis), ECR1^HA^ became exclusively nuclear where it reached maximum levels of expression. ECR1^HA^ was downregulated during budding (Fig. 3C, S/M images) and was absent from G_1_ parasites (Fig. 3C, G_1_ images). Altogether, these results indicate that ECR1 is expressed starting in early S phase through early mitosis.

### Disruption of ECR1 leads to severe morphological defects in mitotic structures.

To further investigate ECR1 function, we introduced the L413H mutation along with a 3×Myc epitope tag by knock-in strategy into the ECR1 gene sequence of a parental strain not previously exposed to chemical mutagenesis (Fig. S1B, diagram). The conversion of native ECR1 into tsECR1^Myc^ generated a temperature-sensitive strain that reproduced the mitotic and cytokinetic defects at high temperature of the original 11-51A1 ts mutant including the disruption of nucleus packaging into daughter buds (Fig. 4A, image 7, and Fig. S1C). At the permissive temperature (34°C), the recreated tsECR1^Myc^ strain replicated similar to the parent strain and possessed healthy centrosome structures (Fig. S1C). Similar to the epitope-tagged native protein (Fig. 3C), tsECR1^Myc^ at 34°C was first detected in the centrocone following duplication of the centrosome outer core (Fig. 4A, images 1 to 3). In normal replicating tachyzoites, the centrocone is closely aligned with the centrosome inner core (Fig. 4A, images 4 to 6) and resolved from the outer core of the centrosome (2). In contrast, centrosome control structures were dramatically affected by the loss of tsECR1^Myc^ at 40°C (Fig. 4A and B). Inner (images 8 to 10) and outer (images 7, 13, and 14) core physical and stoichiometric balance was disrupted, leading to some nuclear packages lacking any associated centrosome core. The changes resulting from the loss of tsECR1^Myc^ at 40°C were not confined to centrosome defects. The usual close alignment of the intranuclear kinetochore (visualized by CenH3) (14) with the centrocone and inner centrosome core was uncoupled, and the CenH3 structure became extranuclear (Fig. 4B). For many nuclear packages, there was no evidence of CenH3 staining present in or nearby the distinct DAPI staining packages (Fig. 4B, images 14 and 20). Finally, the most dramatic change was a complete loss of MORN1 centrocone staining (images 21 and 22), suggesting that the null phenotype of tsECR1^Myc^ at 40°C caused severe defects in the intranuclear organization of the mitotic machinery, including the centrocone and associated kinetochores.

### The ECR1 interactome reveals functions in regulating the parasite cell cycle.

**FIG 5:**
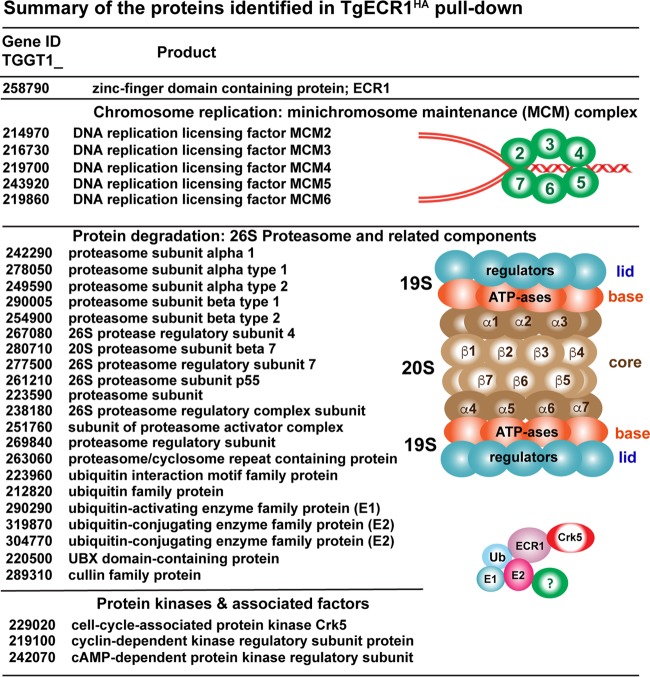
ECR1 proteomics. Proteomic analysis of proteins pulled down with ECR1^HA^ includes five minichromosome maintenance complex proteins (MCM2 to MCM6) required for DNA replication. The ECR1 proteome also contained a number of 20S core alpha and beta subunits, 19S regulatory particle proteins, as well as members of ubiquitination machinery, e.g., ubiquitin-activating enzyme, two ubiquitin-conjugating enzymes. ECR1 also interacted with protein kinases and associated factors, including a CDK-related kinase, TgCrk5 (16). cAMP, cyclic AMP.

In order to better understand the ECR1 mechanism, we performed immunoprecipitation (IP) and liquid chromatography coupled to tandem mass spectrometry (LC-MS/MS) (IP/LC-MS/MS) on wild-type ECR1^HA^ parasites using standard 37°C culture conditions (Fig. 5 and Data Set S1). Purified ECR1^HA^ complexes from whole-cell lysates were resolved by electrophoresis and subjected to mass spectrometry analysis (see Materials and Methods). This experiment identified 123 proteins including ECR1^HA^ itself (24.9% coverage, 47 total spectra) (Data Set S1). Importantly, many nuclear factors were coimmunoprecipitated with ECR1^HA^, including five of six subunits of the minichromosome maintenance complex (MCM_2_ to MCM_7_ [MCM_2-7_]; 1.34%, 69 spectra), which is the essential helicase of semiconservative DNA replication in eukaryotes (15). The ECR1^HA^ pulldown also revealed significant interactions with the ubiquitin-mediated protein degradation machinery (24 proteins) and with 1 (*T*. *gondii* Crk5 [TgCrk5], TGME49_229020) of 10 serine/threonine protein kinases encoded in the *T. gondii* genome that are distantly related to human cyclin-dependent kinases (16). ECR1 interacts with an E1 ubiquitin-activating enzyme, two E2 ubiquitin-conjugating enzymes, UBX domain-containing protein, alpha and beta subunit types, and regulatory subunits of the 26S proteasome (Fig. 5).

10.1128/mBio.00579-17.4 DATA SET S1  Complete proteomic results for Fig. 5 experiments. Download DATA SET S1, DOCX file, 0.2 MB. Copyright © 2017 Naumov et al. 2017 Naumov et al. This content is distributed under the terms of the Creative Commons Attribution 4.0 International license.

**FIG 6:**
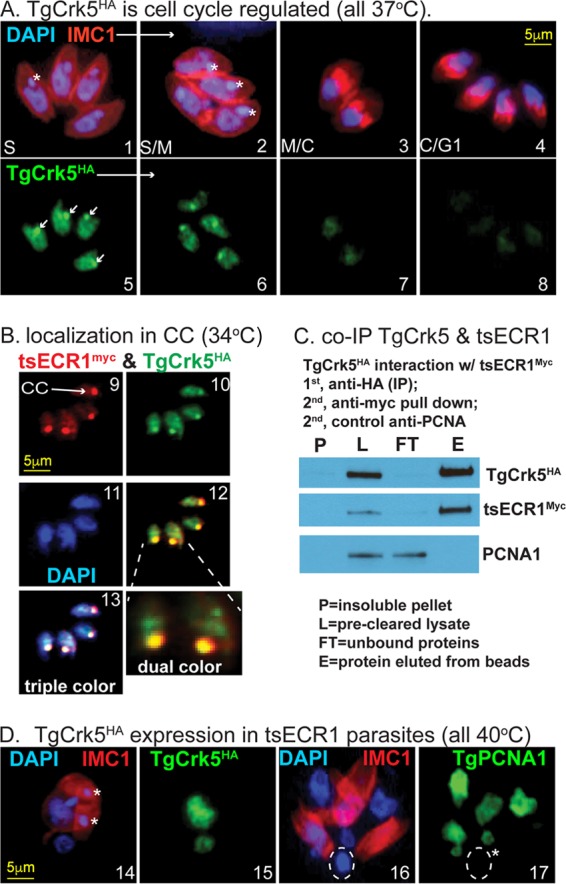
TgCrk5 is associated with ECR1 during tachyzoite replication. (A) TgCrk5^HA^ transgenic parasites were costained with anti-HA, anti-IMC1, and DAPI, and representative images of parasites in S through early G_1_ phase of the next cell cycle are shown. Images 1 to 4 depict the merging of αIMC1 and DAPI staining showing the mother IMC and daughter buds in relation to genomic and plastid DNA. Note the daughter buds formed in image 3 precede nuclear division, whereas nuclei in the four parasites in image 4 are now packaged into the nearly mature daughters representative of late cytokinesis and early G_1_ cell cycle time periods. The white asterisks indicate plastid DNA. Images 5 to 8 depict costaining of the parasites in images 1 to 4 with anti-HA, revealing the cell cycle expression patterns of TgCrk5^HA^. The maximum expression in S phase and low to near undetected expression in late cytokinetic/early G_1_ parasites are consistent with the encoded mRNA cell cycle profile (Fig. 7A). Localization of TgCrk5^HA^ in the centrocone is indicated by white arrows in image 5. (B) TgCrk5 was epitope tagged with 3×HA in tsECR1^Myc^ transgenic parasites. IFA analysis (images 9 to 13) of this new dual-epitope-tagged strain demonstrated colocalization of tsECR1^Myc^ and TgCrk5^HA^ in the parasite centrocone (CC) and nucleus at 34°C. Note that the expression level of TgCrk5^HA^ was higher than that of tsECR1^Myc^ in these parasites. Image 12 depicts merged staining results for TgCrk5^HA^ and tsECR1^Myc^ expression with higher magnification of the two centrocone structures indicated. Image 13 depicts merged staining results for anti-HA, anti-Myc, and DAPI stain used to indicate nuclear DNA. (C) Immunoprecipitation of TgCrk5^HA^ confirms interaction with tsECR1^Myc^ in parasites grown at 34°C. Western analysis reveals that virtually all cellular tsECR1^Myc^ protein was pulled down with TgCrk5^HA^, while the abundant nuclear factor TgPCNA1 was not pulled down. The results shown are representative of three independent replicates of the coimmunoprecipitation experiment. (D) IFA analysis of the dual-tagged TgCrk5^HA^/tsECR1^Myc^ strain at 40°C. Similar to panel A, parasites were costained for DNA and IMC1 (images 14 and 16) and then for TgCrk5^HA^ (image 15) or TgPCNA1 (image 17). The white asterisks in images 14 and 17 indicate plastid DNA, which in images 16 and 17 have aggregated into a single deposit (dashed circle) that does not contain TgPCNA1. The scale bars for image series in panels A, B, and C are indicated once in each series.

**FIG 7:**
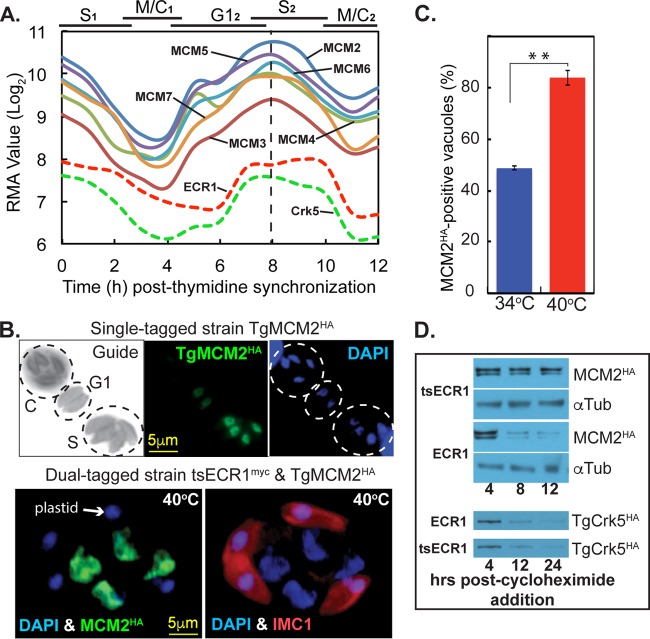
Disruption of ECR1 stabilizes TgMCM2 expression. (A) TgMCM_2-7_ mRNA levels in synchronized tachyzoites revealed nearly identical cyclical profiles (dashed vertical line indicates shared peak expression). All mRNA data from our cell cycle transcriptome were deposited in ToxoDB (http://toxodb.org) (21). Cyclical profiles for ECR1 and TgCrk5 mRNAs are included to show relative cell cycle timing; TgMCM_2-7_ mRNAs peak earlier that ECR1 and TgCrk5 mRNAs. (B) Cell cycle analysis of TgMCM2^HA^ expression. The cells were costained with anti-HA (TgMCM2^HA^ [green]) and DAPI (DNA [blue]). The Guide panel is a noncolorized/inverted IMC1 expression showing three vacuoles in different cell cycle phases as indicated (budding vacuole [C] and late G_1-_ and S-phase vacuoles). TgMCM2^HA^ maximum and minimum expression occurred in S phase and mitosis, respectively, which was consistent with the encoded mRNA cell cycle profiles in panel A. The scale bar is shown once per series of images. (C) IFA analysis of TgMCM2^HA^ tagged in a tsECR1^Myc^ strain at 40°C. Note that masses of nuclear DNA (DAPI [blue]) in the parasite are associated with high levels of TgMCM2^HA^ (green), whereas abnormal buds (IMC1 [red]) contain only plastid DNA (DAPI [blue]). Parasites positive for TgMCM2^HA^ in 100 randomly selected vacuoles nearly doubled when the tsECR1^Myc^ strain was shifted to 40°C for 24 h. At 34°C, the ∼50% of parasites expressing TgMCM2^HA^ correspond to late G_1_ through S phase as shown in panel C. (D) Two dual-epitope-tagged tachyzoite strains (tsECR1^Myc^ with TgMCM2^HA^ or with TgCrk5^HA^) or the single TgMCM2^HA^ strain was shifted to 40°C, treated with 200 μM cycloheximide to block total protein synthesis, and Western analysis of TgMCM2^HA^ and TgCrk5^HA^ protein levels were determined at the indicated times. α-Tubulin (αTub) is naturally stable and included here to demonstrate equal loading. Note that in tsECR1^myc^ parasites at 40°C, TgMCM2^HA^ protein stability was increased, while the stability of TgCrk5^HA^ was unaffected.

CDK protein kinases are key factors in cell cycle checkpoint control (17); therefore, we further investigated TgCrk5 expression in the tachyzoite cell cycle. The TgCrk5 protein was C-terminally epitope tagged with 3×HA by genetic knock-in, and IFA analysis of TgCrk5^HA^ revealed a cell cycle profile similar to that of ECR1 with maximum protein expression in S phase and low to near undetectable levels in G_1_ parasites (Fig. 6A; also see the mRNA profile in Fig. 7A). Like ECR1, TgCrk5^HA^ is a nuclear factor that concentrates in the centrocone where it colocalized with tsECR1^Myc^ in a dual-epitope-tagged strain generated by serial knock-in (Fig. 6B, tsECR1^Myc^ and TgCrk5^HA^, 34°C images). Utilizing the dual-tagged strain, tsECR1^Myc^ interaction with TgCrk5^HA^ at the permissive temperature (34°C) was confirmed by coimmunoprecipitation (co-IP) of whole-cell lysates (Fig. 6C). Finally, the expression of TgCrk5^HA^ in the dual-tagged strain at 40°C was examined revealing TgCrk5^HA^ protein largely remained associated with parasite chromosome material at 40°C (Fig. 6D), although TgCrk5^HA^ centrocone staining was abolished consistent with the disruption of this structure at high temperature. Staining with TgPCNA1 antibodies distinguishes nuclear chromatin from aggregated plastid DNA in these defective parasites (Fig. 6D, images 16 and 17), and as expected, TgCrk5 colocalizes with TgPCNA1 in the tsECR1 parasites (data not shown).

### Loss of tsECR1 affects the stability of TgMCM2.

The interaction of ECR1 with TgMCM proteins (Fig. 5) led us to examine the expression of TgMCM2 in tachyzoites possessing native versus ts mutant ECR1 alleles. In *T*. *gondii* tachyzoites, mRNAs encoding the MCM_2-7_ proteins are cyclically expressed and tightly coordinated with maximum expression in late G_1_ phase (Fig. 7A). In contrast, ECR1 and TgCrk5 mRNA and protein levels were downregulated later in S phase and have broader maximum time of expression (Fig. 3, 4, and 7A). To confirm the periodic expression of representative MCM factors in *T*. *gondii* at the protein level, we epitope tagged TgMCM2 with 3×HA by genetic knock-in and evaluated cell cycle expression of this protein in randomly growing tachyzoites (Fig. 7B). Roughly half of transgenic tachyzoites in a randomly growing population expressed TgMCM2^HA^ with peak expression during the transition from G_1_ into S phase (Fig. 7B) consistent with the encoded mRNA (Fig. 7A). During daughter bud formation, TgMCM2^HA^ protein decreased below IFA detection (Fig. 7B). We then investigated the expression of TgMCM2^HA^ in parasites carrying the tsECR1 mutation (Fig. 7C and D). In these ts mutant parasites, TgMCM2^HA^ was detected at high levels in more than 80% of the population (Fig. 7C) and was colocalized to the masses of chromosome DNA that accumulated in the mother parasite at 40°C (Fig. 7B, bottom panel). The increase in TgMCM2^HA^ levels at 40°C in the tsECR1 parasites was due in part to greater TgMCM2^HA^ stability in tsECR1 parasites (which lack ECR1 at high temperature) compared to parasites that express native ECR1 (Fig. 7D). Protein stabilization was not a generalized phenotype of the loss of tsECR1 or a consequence of high temperature, as there was no increased stabilization of the TgCrk5 partner in tsECR1 parasites grown at 40°C (Fig. 7D) nor did we detect changes in the stability of α-tubulin. It should be noted that we did not detect stable association of MCM2^HA^ with tsECR1^Myc^ at permissive temperatures using co-IP methods that successfully confirmed tsECR1^Myc^/TgCrk5^HA^ interaction in these parasites (results not shown). If MCM2 is an ECR1 substrate, then the interaction is expected to be transient and likely detected only by using substrate-trapping techniques (18).

## DISCUSSION

**FIG 8:**
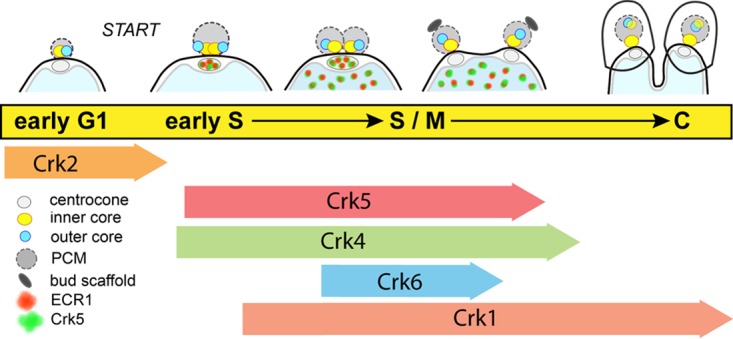
Major control mechanisms of the *T. gondii* tachyzoite cell cycle. Major cell cycle transitions are indicated by conventional phase designations overlaid by the sequential timing of centrosome outer and inner cores and centrocone duplication in dividing tachyzoites. The cell cycle periods corresponding to the mitotic events diagrammed at the top of the figure are linked to the timing of proposed functions of TgCrks indicated below (16). The localization and timing of expression of ECR1 (green) and TgCrk5 (red) that begins in the centrocone and shuttles to the nucleus are indicated. Note that four TgCrks regulating S-phase and mitosis progression are unusual and distinct from well-studied eukaryotic models (yeast and human cells) that require one or two CDKs for these same processes (16). PCM, pericentrosomal matrix surrounding the centrosome cores.

Compared to yeast and mammalian somatic cell proliferation, cell division of apicomplexan asexual life cycle stages is unique in a number of ways. The number of progeny produced from a single mother cell varies widely and is a characteristic of each species and stage, which indicates that parasite genetics is responsible for these differences. The ability to suspend cytokinesis (and nuclear division in some cases, e.g., *Sarcocystis neurona*) (19), while chromosomes and nuclei are reduplicated is remarkable, as is the cytoskeletal complexity that must be assembled to produce infectious daughter parasites. The knowledge that *Apicomplexa* replication is unusual was an early discovery of morphogenic studies, (e.g., reference 4), and we expected the molecular control of apicomplexan cell division to also have distinct features. Through comparative genomics and experimental genetics, major checkpoint mechanisms of the *T. gondii* tachyzoite stage were recently defined (20). For a single-cell eukaryote, there are a large number of Crk protein kinases required for tachyzoite replication, including the ECR1/TgCrk5 complex described here (see the model in Fig. 8) (20). A single TgCrk controls G_1_ progression, while up to four TgCrks regulate karyokinesis and/or cytokinesis in the tachyzoite (16). In contrast to a recent suggestion (21), there are no canonical cyclins (A to E types) in the major *Apicomplexa* families; cyclins in these parasites are atypical, as are many of the TgCrks (16). Oscillating cyclins program checkpoint kinases in higher eukaryotes, however, experimental evidence indicates that some TgCrks lack cyclin partners. The discovery here that TgCrk5 is periodically expressed in the tachyzoite is unusual for a potential checkpoint kinase, and dynamic expression extends to mitotic kinases TgCrk4 and TgCrk6 (16). Perhaps because they are periodic, TgCrk4 and TgCrk6 lack detectable cyclin partners (based on co-IP experiments) (16), and it is possible that the cyclically expressed TgCrk5 will also lack a cyclin partner.

The enzymology of DNA synthesis of eukaryotes is well established and conserved (15). The DNA helix is unwound by an active helicase assembled from six core MCM_2-7_ proteins, and new DNA strands are then produced by DNA polymerases supported by accessory factors (see Fig. S3B in the supplemental material). *Apicomplexa* parasites possess most of this DNA synthetic machinery (Fig. S3A), which includes a requirement for a topoisomerase as we have demonstrated here (Fig. 1). *Toxoplasma* tachyzoites carrying a lethal ts mutation in TgTopo-II show a defective cell cycle phenotype similar to that of tsECR1 at the nonpermissive temperature. However, more than the basic machinery is conserved, as the topology of G_1_-through-S-phase progression in these parasites has parallels to higher eukaryotes. Like other eukaryotes, ordered gene expression (protein synthesis first, DNA replication factors second) in the apicomplexan G_1_ phase (22, 23) prepares the way for DNA synthesis. The ability to synchronize *T. gondii* tachyzoites at the G_1_/S boundary (6) indicates that there is a Start-like checkpoint (likely controlled by TgCrk2) (16) controlling commitment to DNA synthesis, and the faithful reduplication of nuclei in apicomplexans indicates some type of “copy once-only once” rule is also active (Fig. 8). In human cells, the E2F/CDK4,6/retinoblastoma controls the G_1_ pathway (24), and multiple protein regulators beyond the CDK/cyclin mechanisms coordinate each step of chromosome replication (15). Orthologs of these human cell cycle regulators are not present in *Apicomplexa* genomes (Fig. S3A), although there is precedence for lineage-specific proteins to produce similar cell cycle topologies (25). Therefore, the question is not if but what are the cell cycle regulators in the *Apicomplexa*.

10.1128/mBio.00579-17.3 FIG S3  DNA replication factors and major steps in the semiconservative replication of eukaryotic chromosomes. (A) Known factors (yeast and human) involved in eukaryotic chromosome replication were used to probe the translated genome of *T. gondii* and *P. falciparum* (E. Sacco, M. M. Hasan, L. Alberghina, and M. Vanoni, Biotechnol Adv 30:73–98, 2012, https://doi.org/10.1016/j.biotechadv.2011.09.009). Protein factors highlighted in yellow were not found. (B) A model showing the major steps of chromosome replication in eukaryotes with the hypothesized function of ECR1 as an E3 ligase incorporated into the model. Download FIG S3, TIF file, 2.2 MB. Copyright © 2017 Naumov et al. 2017 Naumov et al. This content is distributed under the terms of the Creative Commons Attribution 4.0 International license.

The ubiquitin-proteasome system serves a critical role in the regulation of chromosome replication, and the basic regulatory factors are conserved and active in the *Apicomplexa* (26). A full complement of ubiquitin-conjugating factors and proteasome components are present, and a recent global survey of ubiquitin-conjugated proteins in *T. gondii* tachyzoites (26) determined that ∼35% of proteins with this modification are cell cycle regulated (22). Relevant to this study is a remarkable peak in the ubiquitination and phosphorylation of proteins in late S phase of the tachyzoite cell cycle (26). The RING/TRAF zinc finger factor, ECR1, characterized here may be part of this system (Fig. 8 and Fig. S3B). ECR1 interaction with many components of the proteasome as well as two E2-conjugating enzymes indicates that this factor could be a unique E3 ligase, which will require future biochemical experiments to confirm. Furthermore, ECR1 potential interaction with MCMs along with the stabilization of MCM2 when ECR1 is depleted (Fig. 7) suggests that ECR1 may specifically function in the licensing of chromosome origins of replication in the tachyzoite. The phenotypic similarity of parasites with a tsECR1 allele and those with a tsTgTopo-II allele is consistent with both of these factors functioning in DNA licensing. The timing of ECR1 expression in S phase and early mitosis, which is after the increase in MCM_2-7_ expression in G_1_ (Fig. 7A), suggests that this factor is not responsible for the initial licensing of origins. However, all eukaryotes need a mechanism that prevents relicensing during active chromosome replication. In human cells, the protein geminin serves this function and shares biology similar to that of ECR1 described here. Geminin is active only in S phase through early mitosis and interacts with and is regulated by a CDK-related kinase (human CDK2) through a reversible ubiquitination mechanism (27–29). Like ECR1, geminin has functions beyond regulating chromosome replication that involve ensuring proper centrosome duplication and spindle formation (30). It remains possible that apicomplexan parasites will have a divergent geminin-like protein, and the ECR1 proteome (Data Set S1) includes 24 unknown proteins with 9 proteins encoded by an mRNA that has maximum expression in S phase (gene records for all ECR1 interactors can be obtained at ToxoDB [http://toxodb.org]). These nine proteins also possess coiled-coil domains, which is the key structural feature of human geminin.

The presence of multiple microtubule organizing centers (MTOCs; centrosome/centrocone and apical complex) in apicomplexan parasites (31, 32) sets these parasites apart from well-studied eukaryotic models (yeast and mammalian cells) that have a single MTOC during interphase (33). Our recent discovery of a novel bipartite centrosome with independent core centers that separately regulate cytokinesis and karyokinesis adds further physical and molecular complexity to apicomplexan cell division (2). The centrocone and apical complex MTOCs are conserved across *Apicomplexa* lineages with few if any parallels in better-studied eukaryotes. Contained within the nuclear membrane, the enigmatic centrocone (34) is present throughout apicomplexan cell division (35), and in *T. gondii* tachyzoites, this structure duplicates and then separates starting in late S phase (Fig. 8) (2). The appearance of the ECR1/TgCrk5 complex in the centrocone prior to duplication suggests that these factors may have a role in preparing this structure for mitosis. The mitotic spindle originates in the centrocone (36), and through this structure, nuclear and cytoplasmic mitotic processes are also physically connected (2, 14). Our and other studies show that this involves the centrosome inner core on the cytoplasmic face (2) and the kinetochore on the nuclear side (14). In metaphase of the tachyzoite, these mitotic structures are aligned in a typical linear array with the centrocones anchoring each pole (2). There is clear evidence from studies of other eukaryotes that spindle structures house cell cycle regulatory proteins. Classically, the transitory association of CDK1 and mitotic cyclin complex in the yeast spindle pole body is associated with activation of this checkpoint mechanism (37, 38). In budding and fission yeast, regulatory protein association with the spindle pole body is also required for mechanisms of mitotic exit, which includes factors of the mitotic exit network (MEN) and septation initiation network (SIN) complexes (39, 40). Until our study here, only one protein, MORN1, had been localized within the apicomplexan centrocone, which may be functionally, if not structurally equivalent to the yeast spindle pole body. The MORN1 marker has permitted the morphogenesis of this important structure to be defined in several apicomplexan parasites (35). We have established here that ECR1 and TgCrk5 are new markers of the centrocone, and their presence in the centrocone demonstrates that potential checkpoint controls are housed in this unique spindle organizing structure. The importance of the ECR1/TgCrk5 complex for the duplication of the centrocone and maintaining the physical connection between nuclear and cytoplasmic mitotic centers was revealed in the lethal arrest of the tsECR1 ts mutant (Fig. 4). At high temperatures, inner and outer centrosome cores, the centrocone, and the kinetochores became uncoupled, and all three structures showed reduced or abnormal duplication (Fig. 4). The stability of TgCrk5 is unaffected by the loss of ECR1; however, localization of TgCrk5 in the centrocone requires this factor. Altogether, these results indicate that the localization of ECR1/TgCrk5 in the centrocone and nucleus likely does not represent inactive versus active forms, and instead the results suggest that the ECR1/TgCrk5 complex has multiple temporally regulated functions vital for tachyzoite cell division. The association of this complex prior to centrocone duplication and spindle formation may be required to prepare this structure for mitosis and to establish communication between the newly duplicated centrosome and nuclear kinetochore, while nuclear localization of ECR1/TgCrk5 could be required to prevent relicensing of origins during chromosome replication. There is evidence from phosphoproteomics that ECR1 is phosphorylated, as is TgCrk5 (see ToxoDB gene records), opening the possibility that ECR1 function may require TgCrk5 phosphorylation. Deciphering the full molecular details of the ECR1/TgCrk5 mechanisms will require further molecular dissection as well as understanding what other proteins participate, including the identities of substrates for both the potential ubiquitination and phosphorylation activities. In addition, ECR1 and TgCrk5 provide new tools to probe the scope of critical cell cycle functions housed in the centrocone compartment. While there is more to be done, what this study, and the related investigation of *T. gondii* checkpoints (16) shows, is that our understanding of the molecular basis of apicomplexan cell division is beginning to finally catch up to the remarkable morphogenic processes discovered decades ago.

## MATERIALS AND METHODS

### Parasite cell culture and flow cytometry.

Parasites were grown in human foreskin fibroblasts (HFF) as described previously (41). The temperature-sensitive (ts) mutants 11-51A1 and 13-64D5 were obtained by chemical mutagenesis (7) of the RHΔ*hxgprt* parasite strain (42). All transgenic lines used in this study were produced in the RHΔ*ku80*Δ*hxgprt* strain (43); see Data Set S2 for the full lists of genes, primers, and transgenic strains. Growth measurements were performed using parasites presynchronized by limited invasion as previously described (10, 44). Parasite vacuoles in the infected cultures were evaluated over various time periods with average vacuole sizes determined at each time point from 50 to 100 randomly selected vacuoles.

10.1128/mBio.00579-17.5 DATA SET S2  Transgenic strains and oligonucleotide primers used in this study. Download DATA SET S2, XLSX file, 0.1 MB. Copyright © 2017 Naumov et al. 2017 Naumov et al. This content is distributed under the terms of the Creative Commons Attribution 4.0 International license.

Parasite nuclear DNA content was determined by flow cytometry using propidium iodide (PI) (Sigma, St. Louis, MO) as described previously (6). Briefly, filter-purified tachyzoites were fixed in 70% (vol/vol) ethanol, pelleted at 3,000 × *g*, suspended in phosphate-buffered saline (PBS) (6 × 10^6^ parasites/ml), and stained with PI (final concentration of 0.2 mg/ml in a total volume of 0.5 ml). RNase cocktail (250 U; combination of RNase A, RNase T1) (Ambicon Corp., Austin, TX) was added, and the parasites were incubated in the dark at room temperature for 30 min. Nuclear DNA content was measured based on PI (FL-2) fluorescence using a FACSCalibur flow cytometer (Becton-Dickinson Inc., San Jose, CA). Fluorescence was collected in linear mode (10,000 events), and the results were quantified using CELLQuest v3.0 (Becton-Dickinson Inc.).

### Immunofluorescence assays and Western analysis.

Confluent HFF cultures on glass coverslips were infected with parasites for the indicated times. Infected monolayers were fixed, permeabilized, and incubated with antibody as previously described (10). The following primary antibodies were used; antibody against *T*. *gondii* CenH3 (anti-TgCenH3) rabbit polyclonal (kinetochore stain) (14), antihemagglutinin (anti-HA) rat monoclonal (3F10; Roche Applied Science), anti-Myc rabbit monoclonal (71D10; Cell Signaling Technology), anti-human centrin 2 rabbit polyclonal (centrosome outer core stain) (11), anti-MORN1 rabbit polyclonal (centrocone and basal complex stains kindly provided by Marc-Jan Gubbels, Boston College, MA), anti-IMC1 mouse monoclonal or rabbit polyclonal (parasite shape and internal daughter bud stains kindly provided by Gary Ward, University of Vermont). All Alexa Fluor-conjugated secondary antibodies (Molecular Probes, Life Technologies) were used at a dilution of 1:500. The coverslips were mounted with Aquamount (Thermo Scientific) and viewed on a Zeiss Axiovert microscope equipped with a 100× objective.

For Western analysis, purified parasites were washed in cold PBS and collected by centrifugation. Total lysates were obtained by suspending the parasite pellets with Laemmli loading dye, heated at 75°C for 10 min, and briefly sonicated. After separation on the SDS-polyacrylamide gels, proteins were transferred onto nitrocellulose membranes and probed with the following antibodies: anti-HA rat monoclonal (3F10; Roche Applied Science), anti-Myc rabbit monoclonal (Cell Signaling Technology), and antitubulin mouse polyclonal (12G10; kindly provided by Jacek Gaertig, University of Georgia). After incubation with secondary horseradish peroxidase (HRP)-conjugated antibodies against anti-mouse, anti-rabbit, or anti-rat primary antibodies, proteins were visualized using the Western Lightning Plus-ECL chemiluminescence reagents (Perkin-Elmer).

### Identification of temperature-sensitive mutations.

The ts mutant 11-51A1 was complemented using ToxoSuperCos cosmid library as published previously (7, 10, 11). Briefly, ts mutant parasites were transfected with cosmid library DNA (50 μg DNA/5 × 10^7^ parasites/transfection) in 20 independent electroporations. After two consecutive selections at 40°C, parasites were selected by the combination of high temperature and 1 μM pyrimethamine, and then genomic DNA was isolated for marker rescue (7). The rescued genomic inserts were sequenced in order to map the rescue locus to the *T. gondii* genome (ToxoDB). To resolve the contribution of individual genes in the recovered locus, we transformed the ts mutant 11-51A1 with DNA fragments representing four genes included in the locus: TGME49_258810 (gene 2), TGME49_258800 (gene 3), TGME49_258790 (gene 4), and TGME49_258780 (gene 5) (Fig. 2A). The gene-specific DNA fragments were PCR amplified from genomic DNA from the parental strain RHΔ*hxgprt* (primers used are listed in the Data Set S2) and transfected into 1 × 10^7^ parasites using 6 to 10 μg of purified DNA. Transfected 11-51A1 ts mutant populations were grown at the permissive temperature overnight and then selected at high temperature. Successful genetic rescue was achieved only with amplified genomic DNA spanning the TGME49_258790 gene. The defective gene in ts mutant 13-64D5, which has a phenotype similar to that of ts mutant 11-51A1, was identified by whole-genome sequencing followed by selected fosmid (carrying genomic inserts) complementation using a published strategy (12). Whole-genome DNA libraries for ts mutant 13-64D5 were prepared, sequenced, and analyzed for single nucleotide variation according to published methods (12); sequencing of ts mutant 13-64D5 resulted in >150-fold genome coverage and identified 16 nonsynonymous mutations. Based on the sequencing results, a stepwise fosmid complementation strategy was developed for ts mutant 13-64D5 (Fig. 2B), which revealed the defective gene in 13-64D5 was a *T. gondii* ortholog (TGME49_312230) of eukaryotic DNA topoisomerase II.

### Generation of transgenic tachyzoite strains. (i) Endogenous tagging by genetic knock-in technique.

Selected *T. gondii* proteins were tagged with a triple copy of the HA or Myc tag by previously described genetic knock-in protocols (43). PCR DNA fragments encompassing the 3′ end of the gene of interest (GOI) were used to construct the plasmids pLIC-GOI-HA_3X_/*dhfr*-DHFR-TS, pLIC-GOI-HA_3X_/*dhfr*-HXGPRT, and pLIC-GOI-Myc_3X_/*dhfr*-DHFR-TS, and the constructs were transfected into a RHΔ*ku80*Δ*hxprt* strain deficient in nonhomologous recombination and the hypoxanthine-xanthine-guanine phosphoribosyltransferase (HXGPRT) purine salvage protein (43). The double-tagged transgenic lines were established by sequential selection under alternative drug selection with cloning, and dual-epitope tag expression was verified by immunofluorescence assay (IFA). To reconstruct the tsECR1 phenotype in a clean genetic background, a strain expressing the tsECR1^Myc^ was generated using the same C-terminal knock-in strategy (Fig. S1B). The DNA insert used to build the tsECR1 knock-in plasmid was chemically synthesized (GenScript USA Inc., Piscataway, NJ) to incorporate the L413H mutation along with a 3×Myc epitope tag while also modifying the amino acid coding to prevent recombination events bypassing the ts mutation. Phenotypic regeneration of the new tsECR1 strain was verified by IFA and Western analysis (Fig. S2).

### (ii) Ectopic expression of epitope-tagged ECR1.

The coding sequence of ECR1 was amplified from parental RHΔ*hxgprt* or tsECR1 cDNA libraries (see Data Set S2 for primer design), and the DNA fragments were cloned into the pDEST_gra-Myc_3X_/*sag*-HXGPRT vector by recombination (Gateway cloning; Life Technologies), with the 3×Myc tag fused to the C terminus. Constructs were transformed into the parental RHΔ*hxgprt* strain and selected with mycophenolic acid and xanthine. The resulting ECR1 and tsECR1 clones were evaluated by Western analysis (Fig. S1A).

### Phylogenetic analysis of ECR1 and TgTopoII.

The amino acid sequence of ECR1 was compared to all nonredundant coding sequence (CDS) translations in ncbi.org andEupath.org, and we found strong ECR1 orthologs in the coccidian branch of *Apicomplexa* and divergent orthologs in chromerids and filamentous fungi. Convincing TgTopo-II orthologs where identified in all major eukaryotic groups by pBLAST (ncbi.org). Evolutionary analyses were conducted in Phylogeny.fr (45). The bootstrap consensus tree inferred from 100 replicates represents the evolutionary history of ECR1 and TgTopo-II families analyzed by an Advanced method based on the WAG substitution model (45). To build phylogenetic tree of ECR1-related factors, human PDZRN3 protein containing RING and Sina domains was used as an outgroup.

### Proteomics and validation by coimmunoprecipitation.

The ECR1 protein fused to a 3×HA tag on the C terminus was used for proteomic analysis. ECR1^HA^-expressing tachyzoites were grown at a multiplicity of infection (MOI) of 2 for 26 h at 37°C, and 5.48 × 10^9^ parasites were collected before the lysis to ensure a sufficient sample of S-phase parasites. To prepare the parasite lysate, the parasite pellet was incubated for 60 min at 4°C in lysis buffer (0.1% [vol/vol] Nonidet P-40, 10 mM HEPES [pH 7.4], 150 mM KCl, plus protease inhibitors and phosphatase inhibitors) with rotation, and subjected to six freeze-thaw cycles (snap-freezing in liquid nitrogen bath and thawing ice-water bath) with vortexing for 1 min at 4°C before freezing. The parasite lysate was centrifuged at 12,000 × *g* for 30 min at 4°C, and the clarified lysate was used for coimmunoprecipitation. ECR1^HA^ was immunoprecipitated using mouse monoclonal anti-HA tag magnetic beads (μMACS Anti-HA microbeads; Miltenyi Biotec). The parasite lysate was incubated with anti-HA magnetic beads overnight at 4°C with rotation. After the beads were washed four times with cold wash buffer 1 (150 mM NaCl, 1% NP-40, 0.5% sodium deoxycholate, 0.1% SDS, 50 mM Tris-HCl [pH 8.0]) and once with wash buffer 2 (20 mM Tris-HCl [pH 7.5]) by using μ Column (Miltenyi Biotec) (prewashed with buffer containing 0.1% [vol/vol] Nonidet P-40, 10 mM HEPES [pH 7.4], 150 mM KCl) in the magnetic field of the separator, the bound proteins (co-IP complexes) were eluted from the magnetic beads by applying 50 μl of preheated 95°C hot Laemmli sample buffer to the column, then separated by SDS-PAGE (Mini-Protean TGX 4 to 20%; Bio-Rad), and stained with Coomassie blue (GelCode blue stain reagent; Pierce). Each sample lane was cut into 24 slices and separately analyzed by liquid chromatography coupled to tandem mass spectrometry (LC-MS/MS) as previously described (8). Briefly, proteins reduced and alkylated with Tris(2-carboxyethyl)phosphine (TCEP) and iodoacetamide were digested with trypsin and run sequentially on Acclaim PepMap C_18_ Nanotrap column and PepMapRSLC C_18_ column (Dionex Corp). Raw LC-MS/MS data were collected using Proteome Discoverer 1.2 (Thermo Scientific),proteins were searched in Toxo_Human Combined database using in-house Mascot Protein Search engine (Matrix Science), and the final list was generated in Scaffold 5.5.1 (Proteome Software) with the following filters: 99% protein probability with a minimum of two peptides with >95% peptide probability.

Co-IP of tsECR1^Myc^ and TgCrk5^HA^ was performed as follows. Dual-tagged TgRHΔ*ku80*-tsECR1^Myc^::TgCrk5^HA^ parasites were grown at 34°C for 40 h, and 2 × 10^8^ parasites were collected, lysed, and immunoprecipitated on anti-HA beads (Medical and Biological Laboratories, Japan) as described previously (2). Protein extracts were rotated with beads at room temperature (RT) for 1 h, and washed beads were heated for 10 min at 75°C in Laemmli sample buffer to elute bound proteins. Proteins were separated on Mini-Protean TGX 4 to 20% gels (Bio-Rad), transferred to nitrocellulose membrane, and incubated with anti-Myc and anti-HA antibodies. After incubation with secondary HRP-conjugated antibodies (Jackson ImmunoResearch Lab, West Grove, PA), proteins were visualized using Western Lightning Plus-ECL chemiluminescence reagents (Perkin-Elmer).

## References

[1] Bhagavathula AS, Elnour AA, Shehab A 2016 Alternatives to currently used antimalarial drugs: in search of a magic bullet. Infect Dis Poverty 5:103. doi:10.1186/s40249-016-0196-8. 27809883 PMC5095999

[2] Suvorova ES, Francia M, Striepen B, White MW 2015 A novel bipartite centrosome coordinates the apicomplexan cell cycle. PLoS Biol 13:e1002093. doi:10.1371/journal.pbio.1002093. 25734885 PMC4348508

[3] Gubbels MJ, White M, Szatanek T 2008 The cell cycle and Toxoplasma gondii cell division: tightly knit or loosely stitched? Int J Parasitol 38:1343–1358. doi:10.1016/j.ijpara.2008.06.004. 18703066

[4] Sheffield HG, Melton ML 1968 The fine structure and reproduction of *Toxoplasma gondii*. J Parasitol 54:209–226. doi:10.2307/3276925. 5647101

[5] Radke JR, Striepen B, Guerini MN, Jerome ME, Roos DS, White MW 2001 Defining the cell cycle for the tachyzoite stage of Toxoplasma gondii. Mol Biochem Parasitol 115:165–175. doi:10.1016/S0166-6851(01)00284-5. 11420103

[6] Radke JR, White MW 1998 A cell cycle model for the tachyzoite of *Toxoplasma gondii* using the herpes simplex virus thymidine kinase. Mol Biochem Parasitol 94:237–247. doi:10.1016/S0166-6851(98)00074-7. 9747974

[7] Gubbels MJ, Lehmann M, Muthalagi M, Jerome ME, Brooks CF, Szatanek T, Flynn J, Parrot B, Radke J, Striepen B, White MW 2008 Forward genetic analysis of the apicomplexan cell division cycle in Toxoplasma gondii. PLoS Pathog 4:e36. doi:10.1371/journal.ppat.0040036. 18282098 PMC2242837

[8] Suvorova ES, Croken M, Kratzer S, Ting LM, Conde de Felipe M, Balu B, Markillie ML, Weiss LM, Kim K, White MW 2013 Discovery of a splicing regulator required for cell cycle progression. PLoS Genet 9:e1003305. doi:10.1371/journal.pgen.1003305. 23437009 PMC3578776

[9] Radke JR, Guerini MN, White MW 2000 Toxoplasma gondii: characterization of temperature-sensitive tachyzoite cell cycle mutants. Exp Parasitol 96:168–177. doi:10.1006/expr.2000.4568. 11162367

[10] Suvorova ES, Lehmann MM, Kratzer S, White MW 2012 Nuclear actin-related protein is required for chromosome segregation in Toxoplasma gondii. Mol Biochem Parasitol 181:7–16. doi:10.1016/j.molbiopara.2011.09.006. 21963440 PMC3767130

[11] Suvorova ES, Radke JB, Ting LM, Vinayak S, Alvarez CA, Kratzer S, Kim K, Striepen B, White MW 2013 A nucleolar AAA-NTPase is required for parasite division. Mol Microbiol 90:338–355. doi:10.1111/mmi.12367. 23964771 PMC3902653

[12] Vinayak S, Brooks CF, Naumov A, Suvorova ES, White MW, Striepen B 2014 Genetic manipulation of the Toxoplasma gondii genome by fosmid recombineering. mBio 5:e02021. doi:10.1128/mBio.02021-14. 25467441 PMC4324243

[13] Matsuzawa SI, Reed JC 2001 Siah-1, SIP, and Ebi collaborate in a novel pathway for beta-catenin degradation linked to p53 responses. Mol Cell 7:915–926. doi:10.1016/S1097-2765(01)00242-8. 11389839

[14] Brooks CF, Francia ME, Gissot M, Croken MM, Kim K, Striepen B 2011 Toxoplasma gondii sequesters centromeres to a specific nuclear region throughout the cell cycle. Proc Natl Acad Sci U S A 108:3767–3772. doi:10.1073/pnas.1006741108. 21321216 PMC3048097

[15] Sacco E, Hasan MM, Alberghina L, Vanoni M 2012 Comparative analysis of the molecular mechanisms controlling the initiation of chromosomal DNA replication in yeast and in mammalian cells. Biotechnol Adv 30:73–98. doi:10.1016/j.biotechadv.2011.09.009. 21963686

[16] Alvarez CA, Suvorova ES 2017 Checkpoints of apicomplexan cell division identified in Toxoplasma gondii. PLoS Pathog 13:e1006483. doi:10.1371/journal.ppat.1006483. 28671988 PMC5510908

[17] Bártová I, Koca J, Otyepka M 2008 Functional flexibility of human cyclin-dependent kinase-2 and its evolutionary conservation. Protein Sci 17:22–33. doi:10.1110/ps.072951208. 18042686 PMC2144583

[18] Kim TY, Siesser PF, Rossman KL, Goldfarb D, Mackinnon K, Yan F, Yi X, MacCoss MJ, Moon RT, Der CJ, Major MB 2015 Substrate trapping proteomics reveals targets of the betaTrCP2/FBXW11 ubiquitin ligase. Mol Cell Biol 35:167–181. doi:10.1128/MCB.00857-14. 25332235 PMC4295375

[19] Vaishnava S, Morrison DP, Gaji RY, Murray JM, Entzeroth R, Howe DK, Striepen B 2005 Plastid segregation and cell division in the apicomplexan parasite Sarcocystis neurona. J Cell Sci 118:3397–3407. doi:10.1242/jcs.02458. 16079283

[20] Alvarez CA, Suvorova ES 2017 A checkpoint roadmap for the complex cell division of Apicomplexa parasites. bioRxiv doi:10.1101/104646.

[21] Francia ME, Striepen B 2014 Cell division in apicomplexan parasites. Nat Rev Microbiol 12:125–136. doi:10.1038/nrmicro3184. 24384598

[22] Behnke MS, Wootton JC, Lehmann MM, Radke JB, Lucas O, Nawas J, Sibley LD, White MW 2010 Coordinated progression through two subtranscriptomes underlies the tachyzoite cycle of Toxoplasma gondii. PLoS One 5:e12354. doi:10.1371/journal.pone.0012354. 20865045 PMC2928733

[23] Bozdech Z, Llinás M, Pulliam BL, Wong ED, Zhu J, DeRisi JL 2003 The transcriptome of the intraerythrocytic developmental cycle of Plasmodium falciparum. PLoS Biol 1:E5. doi:10.1371/journal.pbio.0000005. 12929205 PMC176545

[24] Qu Z, Weiss JN, MacLellan WR 2003 Regulation of the mammalian cell cycle: a model of the G1-to-S transition. Am J Physiol Cell Physiol 284:C349–C364. doi:10.1152/ajpcell.00066.2002. 12388094

[25] Cross FR, Buchler NE, Skotheim JM 2011 Evolution of networks and sequences in eukaryotic cell cycle control. Philos Trans R Soc Lond B Biol Sci 366:3532–3544. doi:10.1098/rstb.2011.0078. 22084380 PMC3203458

[26] Silmon de Monerri NC, Yakubu RR, Chen AL, Bradley PJ, Nieves E, Weiss LM, Kim K 2015 The ubiquitin proteome of Toxoplasma gondii reveals roles for protein ubiquitination in cell cycle transitions. Cell Host Microbe 18:621–633. doi:10.1016/j.chom.2015.10.014. 26567513 PMC4968887

[27] Li A, Blow JJ 2005 Cdt1 downregulation by proteolysis and geminin inhibition prevents DNA re-replication in Xenopus. EMBO J 24:395–404. doi:10.1038/sj.emboj.7600520. 15616577 PMC545810

[28] Li A, Blow JJ 2004 Negative regulation of geminin by CDK-dependent ubiquitination controls replication licensing. Cell Cycle 3:443–445. doi:10.4161/cc.3.4.816. 15004531 PMC3604806

[29] Li A, Blow JJ 2004 Non-proteolytic inactivation of geminin requires CDK-dependent ubiquitination. Nat Cell Biol 6:260–267. doi:10.1038/ncb1100. 14767479 PMC2691133

[30] Tachibana KE, Gonzalez MA, Guarguaglini G, Nigg EA, Laskey RA 2005 Depletion of licensing inhibitor geminin causes centrosome overduplication and mitotic defects. EMBO Rep 6:1052–1057. doi:10.1038/sj.embor.7400527. 16179947 PMC1371027

[31] Chen CT, Kelly M, de Leon J, Nwagbara B, Ebbert P, Ferguson DJ, Lowery LA, Morrissette N, Gubbels MJ 2015 Compartmentalized Toxoplasma EB1 bundles spindle microtubules to secure accurate chromosome segregation. Mol Biol Cell 26:4562–4576. doi:10.1091/mbc.E15-06-0437. 26466679 PMC4678015

[32] Francia ME, Dubremetz JF, Morrissette NS 2015 Basal body structure and composition in the apicomplexans Toxoplasma and Plasmodium. Cilia 5:3. doi:10.1186/s13630-016-0025-5. 26855772 PMC4743101

[33] Rüthnick D, Schiebel E 2016 Duplication of the yeast spindle pole body once per cell cycle. Mol Cell Biol 36:1324–1331. doi:10.1128/MCB.00048-16. 26951196 PMC4836218

[34] Dubremetz JF 1973 Ultrastructural study of schizogonic mitosis in the coccidian, Eimeria necatrix (Johnson 1930). J Ultrastruct Res 42:354–376. (In French.) doi:10.1016/S0022-5320(73)90063-4. 4702924

[35] Ferguson DJ, Sahoo N, Pinches RA, Bumstead JM, Tomley FM, Gubbels MJ 2008 MORN1 has a conserved role in asexual and sexual development across the Apicomplexa. Eukaryot Cell 7:698–711. doi:10.1128/EC.00021-08. 18310354 PMC2292627

[36] Sibert GJ, Speer CA 1981 Fine structure of nuclear division and microgametogony of Eimeria nieschulzi Dieben, 1924. Z Parasitenkd 66:179–189. 7324546 10.1007/BF00925725

[37] Decottignies A, Zarzov P, Nurse P 2001 In vivo localisation of fission yeast cyclin-dependent kinase cdc2p and cyclin B cdc13p during mitosis and meiosis. J Cell Sci 114:2627–2640. 11683390 10.1242/jcs.114.14.2627

[38] Alfa CE, Ducommun B, Beach D, Hyams JS 1990 Distinct nuclear and spindle pole body population of cyclin-cdc2 in fission yeast. Nature 347:680–682. doi:10.1038/347680a0. 1699136

[39] Krapp A, Schmidt S, Cano E, Simanis V 2001 S. pombe cdc11p, together with sid4p, provides an anchor for septation initiation network proteins on the spindle pole body. Curr Biol 11:1559–1568. doi:10.1016/S0960-9822(01)00478-X. 11676915

[40] Jaspersen SL, Winey M 2004 The budding yeast spindle pole body: structure, duplication, and function. Annu Rev Cell Dev Biol 20:1–28. doi:10.1146/annurev.cellbio.20.022003.114106. 15473833

[41] Roos DS, Donald RG, Morrissette NS, Moulton AL 1994 Molecular tools for genetic dissection of the protozoan parasite Toxoplasma gondii. Methods Cell Biol 45:27–63. doi:10.1016/S0091-679X(08)61845-2. 7707991

[42] Donald RG, Roos DS 1998 Gene knock-outs and allelic replacements in Toxoplasma gondii: HXGPRT as a selectable marker for hit-and-run mutagenesis. Mol Biochem Parasitol 91:295–305. doi:10.1016/S0166-6851(97)00210-7. 9566522

[43] Huynh MH, Carruthers VB 2009 Tagging of endogenous genes in a Toxoplasma gondii strain lacking Ku80. Eukaryot Cell 8:530–539. doi:10.1128/EC.00358-08. 19218426 PMC2669203

[44] Gaji RY, Behnke MS, Lehmann MM, White MW, Carruthers VB 2011 Cell cycle-dependent, intercellular transmission of Toxoplasma gondii is accompanied by marked changes in parasite gene expression. Mol Microbiol 79:192–204. doi:10.1111/j.1365-2958.2010.07441.x. 21166903 PMC3075969

[45] Dereeper A, Guignon V, Blanc G, Audic S, Buffet S, Chevenet F, Dufayard JF, Guindon S, Lefort V, Lescot M, Claverie JM, Gascuel O 2008 Phylogeny.fr: robust phylogenetic analysis for the non-specialist. Nucleic Acids Res 36:W465–W469. doi:10.1093/nar/gkn180. 18424797 PMC2447785

