# Supplementary figures and images for: The Toxoplasma Centrocone Houses Cell Cycle Regulatory Factors
Source: mBio. 2017 Aug 22;8(4):e00579-17. doi: 10.1128/mBio.00579-17 (PMC5565962; doi:10.1128/mBio.00579-17)

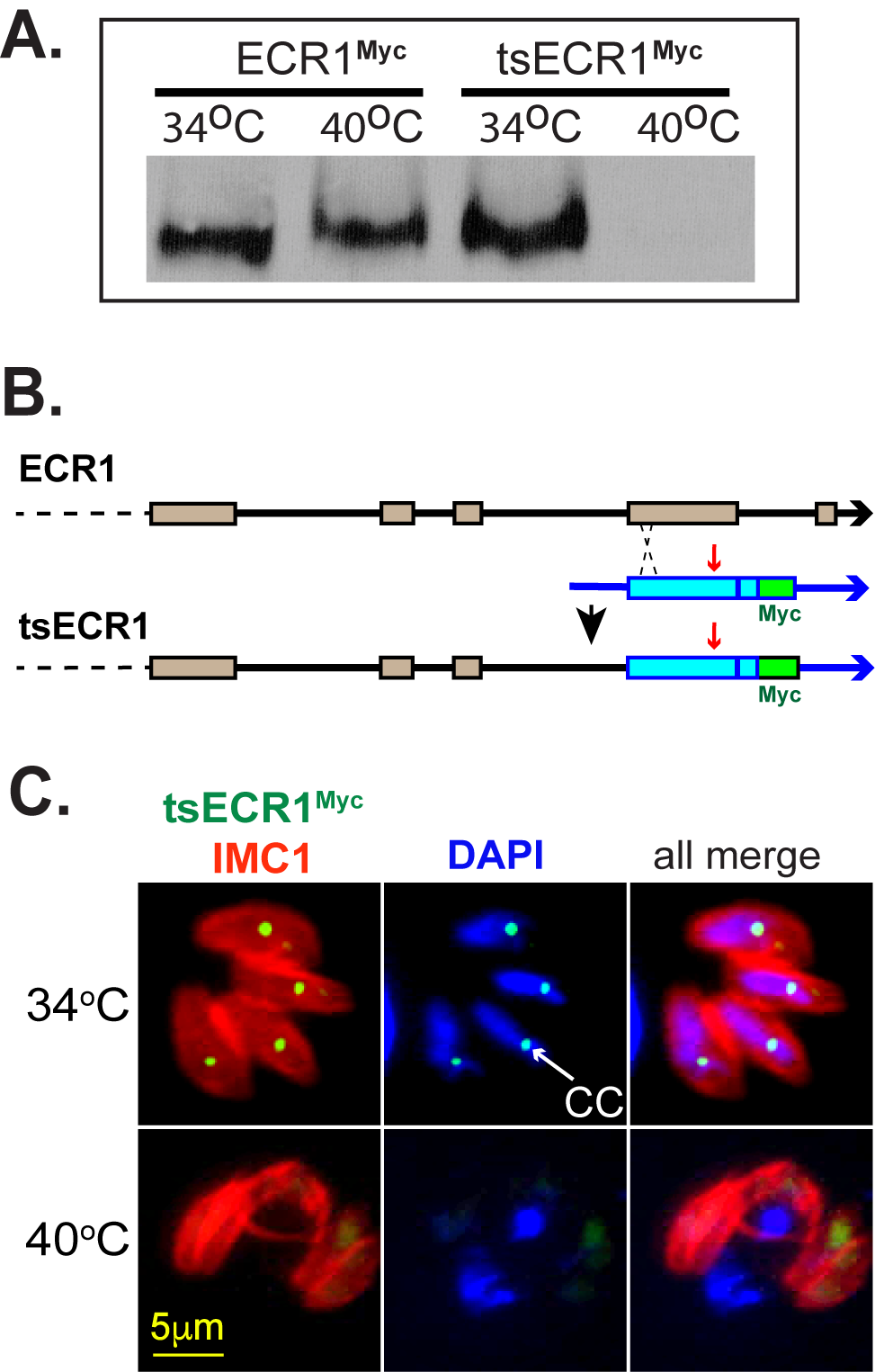

Supplement: FIG S1 [file mbo001173443sf1.tif]

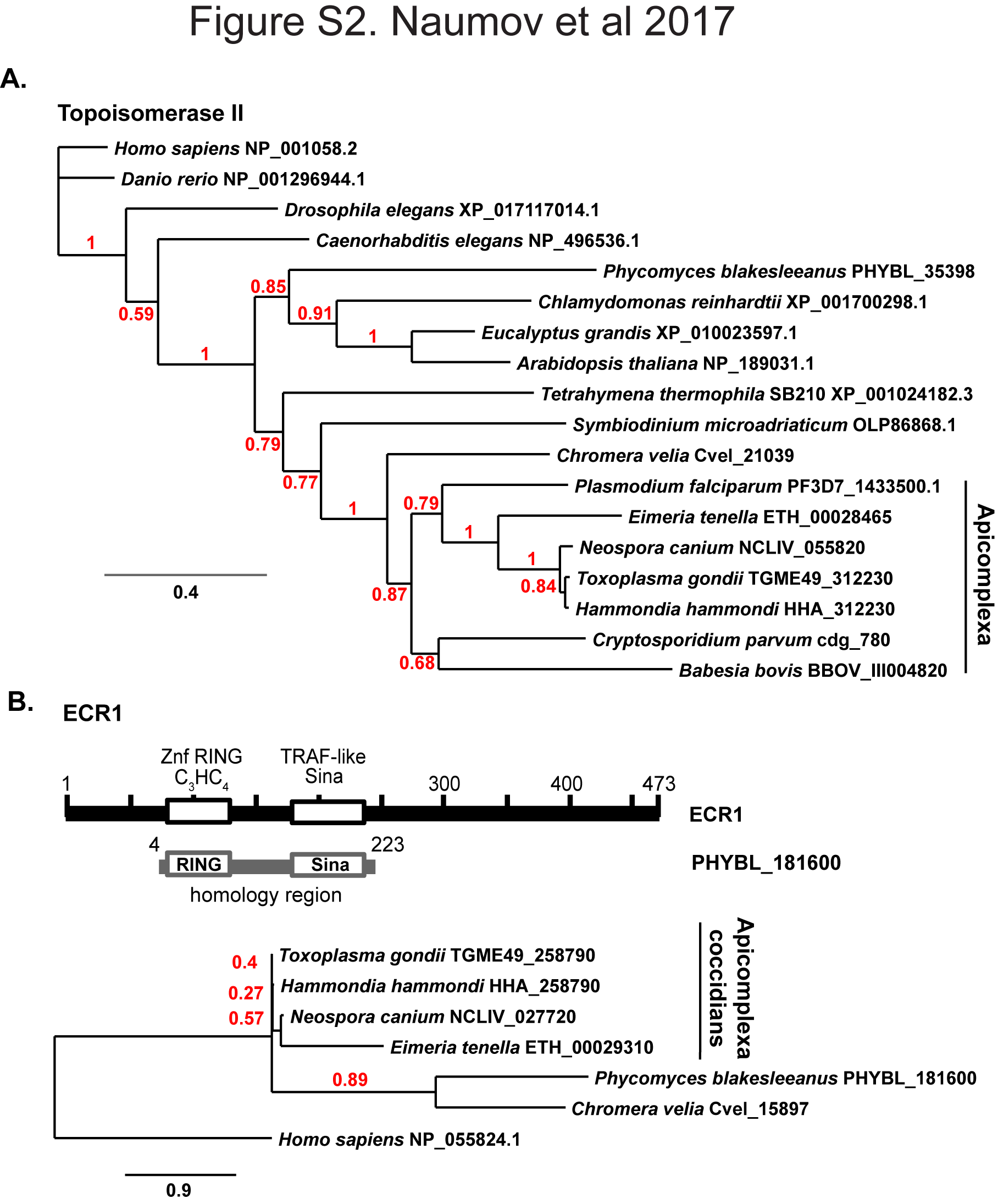

Supplement: FIG S2 [file mbo001173443sf2.tif]

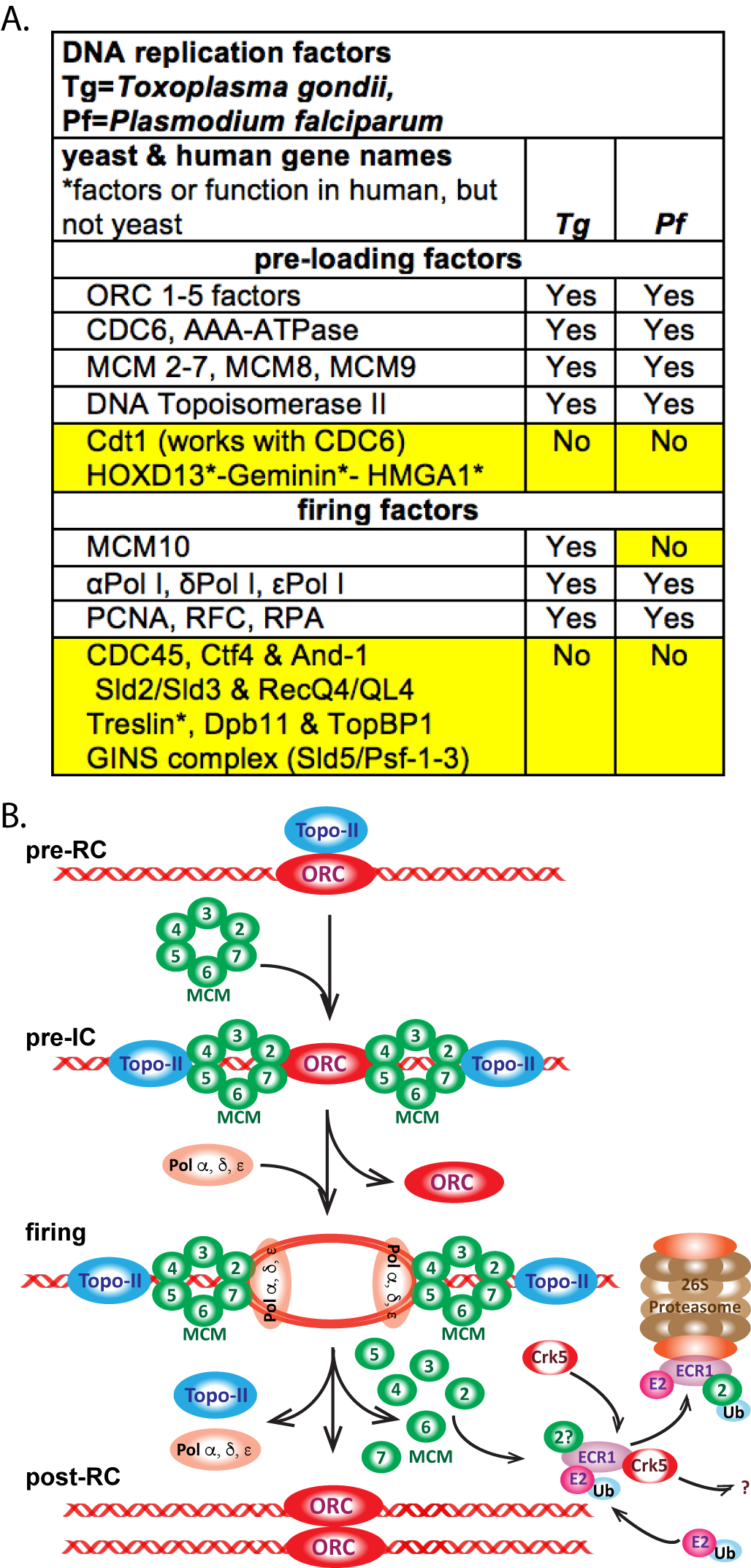

Supplement: FIG S3 [file mbo001173443sf3.tif]
