# Supplementary material for: The Toxoplasma Centrocone Houses Cell Cycle Regulatory Factors
Source: mBio. 2017 Aug 22;8(4):e00579-17. doi: 10.1128/mBio.00579-17 (PMC5565962; doi:10.1128/mBio.00579-17)
Supplement: DATA SET S1 [file mbo001173443sd1.docx]

| **Dataset S1. ECR1-HA proteomics. Top protein hits** | | | | | | |
| --- | --- | --- | --- | --- | --- | --- |
| **Protein accession numbers** | **Protein name** | **Protein molecular weight (Da)** | **Exclusive unique peptide count** | **Exclusive unique spectrum count** | **Total spectrum count** | **Percentage sequence coverage** |
|  | **ECR1-3x HA, pull down target** |  |  |  |  |  |
| TGME49_258790 | ECR1 | 51,907.00 | 13 | 18 | 47 | 24.9% |
|  |  |  |  |  |  |  |
|  | **Chromosome replication** |  |  |  |  |  |
| TGME49_210960 | replication factor C subunit 4, putative | 39,372.80 | 3 | 4 | 4 | 13.40% |
| TGME49_214970 | DNA replication licensing factor MCM2,  putative | 117,246.00 | 16 | 16 | 36 | 19.60% |
| TGME49_216730 | MCM2/3/5 family protein | 113,219.60 | 8 | 9 | 15 | 11.00% |
| TGME49_219700 | DNA replication licensing factor MCM4,  putative | 111,888.40 | 2 | 2 | 2 | 1.78% |
| TGME49_219860 | hypothetical protein; MCM6 DNA replicationlicencing factor | 108,256.70 | 6 | 6 | 8 | 9.16% |
| TGME49_212860 | hypothetical protein; MCM6 DNA replicationlicencing factor | 107,701.70 | 3 | 3 | 6 | 4.63% |
| TGME49_243920 | DNA replication licensing factor MCM5,  putative | 88,213.60 | 2 | 2 | 2 | 2.39% |
| TGME49_219310 | DnaK family protein | 90,142.60 | 17 | 22 | 38 | 24.20% |
| TGME49_226830 | DnaK family protein | 103,193.10 | 10 | 11 | 25 | 14.30% |
| TGME49_290640 | DNA mismatch repair protein MSH6-1,  putative | 171,080.20 | 4 | 4 | 5 | 3.48% |
|  |  |  |  |  |  |  |
|  | **Protein Degradation** |  |  |  |  |  |
| TGME49_212820 | ubiquitin family protein | 119,002.30 | 2 | 2 | 4 | 2.50% |
| TGME49_223590 | proteasome subunit | 39,396.70 | 5 | 6 | 18 | 17.60% |
| TGME49_220500 | UBX domain-containing protein | 43,079.00 | 2 | 2 | 2 | 9.04% |
| TGME49_238180 | 26s proteasome regulatory complex  subunit,putative | 47,552.80 | 2 | 2 | 2 | 6.47% |
| TGME49_239500 | proteasome subunit alpha type, putative | 27,937.50 | 2 | 3 | 8 | 11.10% |

| TGME49_223960 | ubiquitin interaction motif family protein | 41,376.60 | 7 | 9 | 14 | 25.50% |
| --- | --- | --- | --- | --- | --- | --- |
| TGME49_242290 | proteasome subunit alpha1, putative | 28,114.30 | 2 | 2 | 4 | 8.70% |
| TGME49_249590 | proteasome subunit alpha type 5-2, | 28,279.10 | 4 | 5 | 17 | 18.20% |
| TGME49_251760 | subunit of proteaseome activator  complex, putative | 30,712.10 | 2 | 2 | 2 | 12.60% |
| TGME49_254900 | proteasome subunit beta type 2, putative | 22,002.50 | 4 | 4 | 7 | 13.10% |
| TGME49_258150 | proteasome subunit alpha type 7,  putative | 27,080.50 | 4 | 4 | 7 | 15.90% |
| TGME49_261210 | 26s proteasome subunit p55, putative | 55,051.40 | 4 | 5 | 7 | 12.80% |
| TGME49_263060 | Proteasome/cyclosome repeat-containing  protein | 125,595.80 | 20 | 28 | 57 | 22.20% |
| TGME49_267080 | 26S protease regulatory subunit 4,  putative | 49,122.70 | 3 | 3 | 5 | 10.90% |
| TGME49_269840 | proteasome regulatory subunit | 35,261.40 | 4 | 7 | 9 | 20.10% |
| TGME49_277500 | 26S proteasome regulatory subunit 7,  putative | 52,369.70 | 2 | 2 | 6 | 7.77% |
| TGME49_278050 | proteasome subunit alpha type 1,  putative | 27,240.90 | 4 | 4 | 6 | 21.50% |
| TGME49_280710 | 20S proteasome subunit beta 7, putative | 30,266.50 | 2 | 2 | 5 | 10.50% |
| TGME49_289310 | cullin family protein | 103,667.50 | 2 | 2 | 2 | 2.29% |
| TGME49_290005 | proteasome subunit beta type 1, putative | 39,916.60 | 3 | 3 | 7 | 12.80% |
| TGME49_290290 | ubiquitin-activating enzyme E1 family  protein | 119,811.10 | 10 | 10 | 16 | 12.50% |
| TGME49_304770 | ubiquitin-conjugating enzyme subfamily  protein | 17,623.20 | 2 | 2 | 2 | 21.90% |
| TGME49_319870 | ubiquitin-conjugating enzyme subfamily  protein | 35,025.40 | 2 | 2 | 2 | 14.50% |
|  |  |  |  |  |  |  |
|  | **Protein kinases & associated factors** |  |  |  |  |  |
| TGME49_219100 | cyclin-dependent kinase regulatory  subunit protein | 65,347.10 | 2 | 2 | 3 | 4.08% |
| TGME49_229020 | cell-cycle-associated protein kinase CDK,  putative | 138,007.60 | 6 | 7 | 14 | 4.19% |
| TGME49_242070 | cAMP-dependent protein kinase  regulatory subunit | 42,797.40 | 2 | 2 | 2 | 5.19% |

|  |  |  |  |  |  |  |
| --- | --- | --- | --- | --- | --- | --- |
|  | **Gene expression factors** |  |  |  |  |  |
| TGME49_202690 | DNA-directed RNA polymerase II RPB9 | 28,169.70 | 2 | 2 | 2 | 12.40% |
| TGME49_205010 | U2 small nuclear ribonucleoprotein family  protein, putative | 150,785.40 | 6 | 7 | 13 | 5.56% |
| TGME49_221670 | transcriptional elongation factor FACT140 | 134,601.50 | 11 | 13 | 20 | 12.80% |
| TGME49_221950 | splicesome-associated protein, putative | 64,913.30 | 2 | 2 | 3 | 3.94% |
| TGME49_258210 | DNA-directed RNA polymerase II RPB2 | 140,952.40 | 2 | 2 | 2 | 1.51% |
| TGME49_310950 | AP2 domain transcription factor AP2XI-3 | 135,097.20 | 4 | 5 | 6 | 4.79% |
| TGME49_313810 | transcription initiation factor TFIID  complex subunit TAF8 | 66,966.00 | 3 | 3 | 4 | 9.86% |
| TGME49_314830 | pre-mRNA splicing factor subunit,  putative | 12,178.40 | 3 | 3 | 4 | 25.00% |
| TGME49_318260 | transcription initiation factor TFIID subunit  TAF5 | 117,951.50 | 3 | 3 | 3 | 5.67% |
|  |  |  |  |  |  |  |
|  | **Other nuclear proteins** |  |  |  |  |  |
| TGME49_205580 | nuclear factor NF4 | 51,221.00 | 2 | 2 | 2 | 5.63% |
| TGME49_222380 | importin-beta N-terminal domain-  containing protein | 129,491.90 | 2 | 2 | 2 | 1.83% |
| TGME49_223540 | importin-beta N-terminal domain-  containing protein | 122,223.10 | 2 | 2 | 2 | 1.74% |
| TGME49_227970 | histone family DNA-binding protein | 25,188.10 | 2 | 3 | 4 | 14.00% |
| TGME49_233350 | nuclear transport factor 2, putative | 13,832.80 | 2 | 4 | 7 | 32.00% |
| TGME49_233020 | Not1 N-terminal domain, CCR4-Not complex component protein | 83,855.70 | 2 | 2 | 3 | 3.98% |
| TGME49_244110 | nucleosome assembly protein (nap)  protein | 48,587.80 | 2 | 2 | 2 | 5.54% |
| TGME49_273870 | SWI2/SNF2 ISWI-like (AT hook) | 175,266.70 | 2 | 2 | 2 | 1.61% |
| TGME49_288630 | nucleosome assembly protein (NAP),  putative | 32,081.10 | 5 | 7 | 18 | 18.10% |

| **Dataset S1. ECR1 Proteomics. Other protein hits** | | | | | | |
| --- | --- | --- | --- | --- | --- | --- |
| **Protein accession numbers** | **Protein name** | **Protein molecular weight (Da)** | **Exclusive unique peptide count** | **Exclusive unique spectrum count** | **Total spectrum count** | **Percentage sequence coverage** |
| TGME49_221320 | acetyl-CoA carboxylase ACC1 | 287,683.30 | 14 | 16 | 20 | 7.39% |
| TGME49_226730 | aconitate hydratase ACN/IRP | 114,886.90 | 5 | 5 | 11 | 5.31% |
| TGME49_223390 | activating signal cointegrator 1 complex  subunit 3 family 1 ASCC3L1, putative | 248,405.90 | 10 | 10 | 13 | 3.76% |
| TGME49_313670 | adaptin n terminal region domain-containing  protein | 107,008.30 | 3 | 4 | 5 | 4.46% |
| TGME49_221522 | adaptor complexes medium subunit family  protein | 61,126.70 | 2 | 3 | 3 | 3.50% |
| TGME49_225050 | adenosylhomocysteinase, putative | 64,107.10 | 9 | 13 | 23 | 19.70% |
| TGME49_218820 | alba 2 | 15,392.20 | 3 | 3 | 14 | 20.30% |
| TGME49_254880 | Alpha-galactosidase | 81,795.60 | 2 | 2 | 2 | 3.21% |
| TGME49_224350 | aminopeptidase N, putative | 156,764.80 | 6 | 7 | 14 | 4.86% |
| TGME49_310080 | AMP-binding enzyme domain-containing  protein | 87,850.20 | 6 | 7 | 9 | 6.08% |
| TGME49_312110 | apicoplast-associated thioredoxin family  protein Atrx1 | 86,835.40 | 2 | 2 | 2 | 4.68% |
| TGME49_270690 | arginyl-tRNA synthetase | 130,258.90 | 2 | 2 | 2 | 2.81% |
| TGME49_210840 | arginyl-tRNA synthetase family protein | 67,019.60 | 2 | 2 | 3 | 4.20% |
| TGME49_294770 | Armadillo/beta-catenin family repeat-  containing protein | 74,145.90 | 2 | 2 | 2 | 3.66% |
| TGME49_202530 | aspartate-tRNA ligase | 86,804.70 | 2 | 2 | 2 | 3.23% |
| TGME49_246550 | aspartyl protease ASP3 | 69,157.60 | 2 | 2 | 2 | 5.13% |
| TGME49_223840 | ATP-citrate lyase, putative | 139,837.50 | 3 | 3 | 3 | 2.89% |
| TGME49_233520 | ATP-dependent RNA helicase | 88,866.90 | 4 | 5 | 6 | 6.49% |
| TGME49_249180 | bifunctional dihydrofolate reductase-  thymidylate synthase | 68,752.40 | 2 | 3 | 3 | 4.75% |

| TGME49_230450 | bifunctional GMP synthase/glutamine  amidotransferase protein | 62,294.70 | 2 | 3 | 4 | 4.92% |
| --- | --- | --- | --- | --- | --- | --- |
| TGME49_263580 | bromodomain-containing protein | 76,520.20 | 3 | 4 | 4 | 4.76% |
| TGME49_305860 | calcium-dependent protein kinase CDPK3 | 60,431.20 | 1 | 1 | 3 | 5.77% |
| TGME49_249240 | calmodulin, putative | 16,805.20 | 8 | 12 | 31 | 53.00% |
| TGME49_215260 | carbamoylphosphate synthetase | 187,039.40 | 20 | 21 | 33 | 14.40% |
| TGME49_263530 | chaperonin , putative | 13,862.20 | 2 | 3 | 4 | 17.30% |
| TGME49_240600 | chaperonin Cpn60, mitochondrial precursor,  putative | 72,237.80 | 6 | 7 | 11 | 11.40% |
| TGME49_273960 | chaperonin GroS protein | 39,951.20 | 2 | 2 | 4 | 7.75% |
| TGME49_268890 | citrate synthase I | 60,382.90 | 2 | 2 | 2 | 5.18% |
| TGME49_275690 | ClpB, putative | 114,553.70 | 5 | 5 | 7 | 7.05% |
| TGME49_203390 | CRAL/TRIO domain-containing protein | 50,481.30 | 2 | 3 | 5 | 7.24% |
| TGME49_299210 | CTP synthase | 70,997.70 | 4 | 4 | 4 | 8.28% |
| TGME49_232370 | CW-type Zinc Finger protein | 118,715.00 | 2 | 2 | 2 | 1.56% |
| TGME49_310030 | cyclase-associated protein, putative | 22,483.40 | 5 | 8 | 14 | 27.60% |
| TGME49_230520 | cyclophilin 1, putative | 26,224.00 | 3 | 3 | 4 | 11.00% |
| TGME49_219750 | cytochrome c, putative | 12,453.80 | 4 | 6 | 9 | 40.90% |
| TGME49_219540 | cytosolic tRNA-Ala synthetase | 139,519.30 | 22 | 27 | 62 | 21.10% |
| TGME49_270250 | dense granule protein GRA1 | 20,225.10 | 4 | 8 | 17 | 15.30% |
| TGME49_254720 | dense granule protein GRA8 | 28,627.70 | 2 | 2 | 2 | 7.81% |
| TGME49_259550 | dihydropteroate synthase | 82,685.90 | 3 | 4 | 6 | 4.55% |
| TGME49_321620 | dynamin-related protein DRPB | 95,933.40 | 5 | 6 | 11 | 7.67% |
| TGME49_214180 | ENTH domain-containing protein | 65,929.40 | 2 | 2 | 2 | 6.15% |
| TGME49_223410 | eukaryotic initiation factor-4E, putative | 25,981.40 | 4 | 4 | 10 | 13.30% |
| TGME49_288500 | FAD Malate-dehydrogenase (MDH-FAD) | 60,382.40 | 5 | 5 | 6 | 7.09% |
| TGME49_202840 | FHA domain-containing protein | 106,105.50 | 9 | 10 | 25 | 14.10% |
| TGME49_287980 | FHA domain-containing protein | 118,212.30 | 8 | 8 | 8 | 10.40% |

| TGME49_247510 | fructose-bisphospatase II | 42,398.40 | 6 | 6 | 9 | 10.80% |
| --- | --- | --- | --- | --- | --- | --- |
| TGME49_278830 | glucose-6-phosphate 1-dehydrogenase | 98,283.10 | 8 | 8 | 21 | 12.40% |
| TGME49_263870 | glutamate-tRNA ligase | 95,129.60 | 5 | 6 | 10 | 4.71% |
| TGME49_217460 | glutaminyl-tRNA synthetase (GlnRS) | 95,529.60 | 9 | 9 | 1421 | 14.80% |
| TGME49_310670 | glycogen phosphorylase 1, putative | 105,539.20 | 2 | 2 | 2 | 2.85% |
| TGME49_256990 | glycyl-tRNA synthetase | 86,375.20 | 12 | 12 | 17 | 16.20% |
| TGME49_214350 | GTP-binding protein, putative | 44,547.10 | 2 | 2 | 2 | 7.83% |
| TGME49_204160 | GYF domain-containing protein | 189,981.60 | 2 | 2 | 2 | 1.21% |
| TGME49_229180 | HEAT repeat-containing protein | 136,166.40 | 2 | 2 | 3 | 2.42% |
| TGME49_292920 | heat shock protein 75, putative | 95,161.50 | 5 | 5 | 8 | 9.41% |
| TGME49_265450 | hexokinase | 51,497.60 | 8 | 9 | 21 | 19.40% |
| TGME49_251680 | histamine-releasing factor, putative | 19,172.70 | 3 | 4 | 8 | 10.50% |
| TGME49_280600 | histidyl-tRNA synthetase (HisRS), putative | 134,877.60 | 9 | 11 | 13 | 9.27% |
| TGME49_243440 | histone lysine acetyltransferase GCN5-B | 111,546.90 | 2 | 2 | 2 | 4.94% |
| TGME49_243580 | Hit family protein involved in cell-cycle  regulation, putative | 30,516.40 | 2 | 3 | 3 | 11.20% |
| TGME49_203780 | hypothetical protein | 145,087.00 | 4 | 4 | 4 | 3.46% |
| TGME49_209600 | hypothetical protein | 50,201.00 | 5 | 5 | 10 | 12.40% |
| TGME49_211030 | hypothetical protein | 22,215.60 | 5 | 6 | 23 | 32.50% |
| TGME49_212090 | hypothetical protein | 70,186.30 | 14 | 19 | 50 | 29.00% |
| TGME49_225200 | hypothetical protein | 179,692.90 | 2 | 2 | 2 | 1.16% |
| TGME49_226980 | hypothetical protein | 25,353.40 | 2 | 2 | 4 | 14.10% |
| TGME49_232150 | hypothetical protein | 107,552.80 | 2 | 2 | 3 | 2.46% |
| TGME49_233140 | hypothetical protein | 18,161.70 | 3 | 3 | 7 | 19.40% |
| TGME49_237180 | hypothetical protein | 29,156.10 | 10 | 19 | 51 | 39.60% |
| TGME49_241170 | hypothetical protein | 139,692.00 | 2 | 2 | 3 | 1.88% |
| TGME49_243200 | hypothetical protein | 133,446.20 | 3 | 3 | 3 | 3.10% |
| TGME49_244460 | hypothetical protein | 127,300.20 | 4 | 4 | 5 | 5.57% |
| TGME49_249780 | hypothetical protein | 35,575.30 | 3 | 3 | 7 | 17.40% |
| TGME49_249990 | hypothetical protein | 92,588.40 | 2 | 2 | 2 | 3.58% |
| TGME49_253820 | hypothetical protein | 25,221.80 | 2 | 2 | 10 | 11.90% |
| TGME49_258850 | hypothetical protein | 32,751.80 | 4 | 4 | 6 | 10.90% |
| TGME49_261450 | hypothetical protein | 119,311.60 | 8 | 11 | 17 | 8.64% |

| TGME49_268760 | hypothetical protein | 31,988.80 | 7 | 14 | 43 | 21.90% |
| --- | --- | --- | --- | --- | --- | --- |
| TGME49_269950 | hypothetical protein | 104,680.80 | 4 | 4 | 5 | 6.04% |
| TGME49_273905 | hypothetical protein | 57,446.30 | 2 | 2 | 2 | 4.05% |
| TGME49_279380 | hypothetical protein | 34,769.10 | 5 | 5 | 10 | 20.30% |
| TGME49_314260 | hypothetical protein | 23,795.70 | 3 | 3 | 16 | 19.70% |
| TGME49_315270 | hypothetical protein | 84,599.60 | 5 | 5 | 6 | 9.49% |
| TGME49_323100 | hypothetical protein | 15,886.10 | 1 | 2 | 4 | 15.20% |
| TGME49_313140 | isocitrate dehydrogenase | 68,296.90 | 2 | 2 | 3 | 3.70% |
| TGME49_227560 | IWS1 C-terminus protein | 54,332.50 | 6 | 10 | 25 | 11.10% |
| TGME49_271810 | lanp, putative | 31,931.70 | 4 | 4 | 5 | 23.00% |
| TGME49_290670 | leucyl aminopeptidase LAP | 83,237.20 | 11 | 13 | 24 | 12.70% |
| TGME49_236570 | lysine decarboxylase family protein | 43,954.80 | 3 | 5 | 6 | 11.10% |
| TGME49_286750 | MA3 domain-containing protein | 58,971.00 | 4 | 4 | 5 | 6.80% |
| TGME49_290040 | macrophage migration inhibitory factor,  putative | 12,254.40 | 5 | 5 | 13 | 32.80% |
| TGME49_318430 | malate dehydrogenase MDH | 54,761.60 | 12 | 14 | 30 | 30.70% |
| TGME49_289300 | methionyl-tRNA synthetase | 106,101.70 | 3 | 3 | 4 | 3.89% |
| TGME49_269250 | Mov34/MPN/PAD-1 family protein | 38,438.90 | 5 | 6 | 11 | 22.20% |
| TGME49_261000 | MutS domain V domain-containing protein | 102,265.60 | 3 | 3 | 3 | 3.31% |
| TGME49_205558 | NAC domain-containing protein | 20,506.50 | 5 | 8 | 25 | 42.30% |
| TGME49_257090 | NAC domain-containing protein | 38,782.30 | 4 | 7 | 11 | 20.10% |
| TGME49_288830 | NADH dehydrogenase (NDH2-II) | 72,109.60 | 3 | 3 | 5 | 5.63% |
| TGME49_277240 | NTPase I | 22,641.40 | 1 | 1 | 6 | 22.70% |
| TGME49_277270 | NTPase II | 37,376.10 | 3 | 3 | 6 | 14.40% |
| TGME49_295350 | nucleoside diphosphate kinase, putative | 17,639.10 | 6 | 9 | 19 | 47.70% |
| TGME49_259660 | orotate phosphoribosyltransferase | 31,594.30 | 2 | 3 | 4 | 11.70% |
| TGME49_278660 | P-type ATPase4, putative | 144,648.50 | 2 | 2 | 2 | 2.54% |
| TGME49_253900 | parasite porphobilinogen synthase PBGS | 71,375.40 | 2 | 2 | 2 | 3.95% |
| TGME49_207770 | PCI domain-containing protein | 55,464.70 | 7 | 7 | 14 | 17.80% |
| TGME49_227960 | PCI domain-containing protein | 55,709.60 | 6 | 6 | 11 | 9.29% |

| TGME49_292220 | PCI domain-containing protein | 61,441.50 | 9 | 11 | 35 | 22.40% |
| --- | --- | --- | --- | --- | --- | --- |
| TGME49_289650 | PEP-carboxykinase I | 75,344.00 | 7 | 9 | 15 | 10.60% |
| TGME49_214490 | peptidase M16 inactive domain-containing  protein | 148,866.70 | 3 | 4 | 5 | 3.33% |
| TGME49_227948 | peptidase M16 inactive domain-containing  protein | 142,766.80 | 2 | 2 | 2 | 3.75% |
| TGME49_216450 | peptidase, T1 family protein | 27,939.10 | 3 | 3 | 3 | 10.00% |
| TGME49_309210 | peroxiredoxin 6, putative | 44,035.90 | 6 | 6 | 22 | 14.70% |
| TGME49_306960 | phenylalanine--tRNA ligase, beta subunit  protein | 74,585.50 | 3 | 3 | 4 | 7.11% |
| TGME49_297060 | phosphoglycerate mutase PGMII | 38,159.40 | 4 | 4 | 7 | 15.90% |
| TGME49_218780 | phosphoserine aminotransferase, putative | 52,755.10 | 4 | 4 | 10 | 14.10% |
| TGME49_222900 | phosphoserine phosphatase | 190,476.00 | 2 | 2 | 2 | 1.31% |
| TGME49_280380 | poly(ADP-ribose) glycohydrolase | 61,838.00 | 3 | 3 | 3 | 8.32% |
| TGME49_257490 | prefoldin subunit superfamily protein | 33,032.50 | 2 | 2 | 2 | 10.40% |
| TGME49_279390 | proliferation-associated protein 2G4,  putative | 50,341.60 | 4 | 4 | 12 | 10.60% |
| TGME49_311310 | protein phosphatase 2B catalytic subunit,  calcineurin family phosphatse superfamily  protein | 56,447.60 | 2 | 2 | 4 | 3.99% |
| TGME49_270320 | protein phosphatase 2C domain-containing  protein | 58,673.90 | 3 | 4 | 4 | 12.10% |
| TGME49_206610 | pyruvate dehydrogenase complex subunit  PDH-E2 | 96,878.90 | 2 | 3 | 3 | 4.09% |
| TGME49_305980 | pyruvate dehydrogenase complex subunit  PDH-E3I | 66,838.60 | 2 | 2 | 3 | 4.23% |
| TGME49_206470 | pyruvate dehydrogenase complex subunit  PDH-E3II | 55,121.20 | 9 | 10 | 12 | 24.30% |
| TGME49_256760 | pyruvate kinase PyK1 | 57,529.40 | 8 | 16 | 31 | 18.50% |
| TGME49_231170 | RecF/RecN/SMC N terminal domain-  containing protein | 170,448.80 | 2 | 2 | 2 | 1.64% |
| TGME49_291960 | rhoptry kinase family protein ROP40  (incomplete catalytic triad) | 57,948.00 | 2 | 2 | 8 | 4.00% |
| TGME49_291960 | rhoptry kinase family protein ROP40  (incomplete catalytic triad) | 57,948.00 | 2 | 2 | 3 | 4.00% |
| TGME49_310010 | rhoptry neck protein RON1 | 127,433.10 | 10 | 16 | 43 | 11.80% |
| TGME49_262730 | rhoptry protein ROP16 | 76,216.40 | 2 | 2 | 2 | 6.08% |
| TGME49_243730 | rhoptry protein ROP9 | 37,668.20 | 7 | 7 | 13 | 25.50% |
| TGME49_294640 | ribonucleoside-diphosphate reductase large  chain | 98,181.00 | 7 | 8 | 12 | 10.80% |

| TGME49_264610 | RNA recognition motif-containing protein | 44,974.40 | 4 | 5 | 20 | 13.30% |
| --- | --- | --- | --- | --- | --- | --- |
| TGME49_290660 | RNA recognition motif-containing protein | 48,394.90 | 3 | 4 | 7 | 8.07% |
| TGME49_291930 | RNA recognition motif-containing protein | 73,118.90 | 5 | 5 | 20 | 9.08% |
| TGME49_240690 | S-adenosyl methionine synthetase | 45,893.60 | 2 | 2 | 2 | 4.82% |
| TGME49_211670 | S1 RNA binding domain-containing protein | 42,217.30 | 4 | 5 | 8 | 14.30% |
| TGME49_216650 | S15 sporozoite-expressed protein | 48,988.90 | 4 | 5 | 8 | 13.30% |
| TGME49_285870 | SAG-related sequence SRS20A | 34,944.50 | 2 | 2 | 2 | 8.54% |
| TGME49_308840 | SAG-related sequence SRS51 | 50,083.90 | 2 | 3 | 7 | 5.72% |
| TGME49_308020 | SAG-related sequence SRS57 | 41,971.30 | 2 | 2 | 4 | 5.45% |
| TGME49_291680 | Sec23/Sec24 trunk domain-containing  protein | 87,611.30 | 4 | 4 | 4 | 6.07% |
| TGME49_234190 | serine hydroxymethyltransferase 2, putative | 65,116.60 | 4 | 4 | 14 | 7.39% |
| TGME49_231850 | serine-threonine phosophatase 2C (PP2C) | 36,790.40 | 6 | 7 | 16 | 23.00% |
| TGME49_256050 | signal recognition particle 14kd protein | 18,964.40 | 2 | 2 | 2 | 14.50% |
| TGME49_275802 | SRP72 RNA-binding domain-containing  protein | 79,712.30 | 2 | 2 | 3 | 6.44% |
| TGME49_316310 | superoxide dismutase | 22,550.70 | 2 | 3 | 12 | 13.40% |
| TGME49_246500 | surp module domain-containing protein | 71,701.50 | 4 | 4 | 4 | 10.20% |
| TGME49_286920 | SWIRM domain-containing protein | 83,974.90 | 5 | 5 | 10 | 8.97% |
| TGME49_261010 | tat-binding family protein, putative | 46,073.90 | 3 | 3 | 6 | 12.00% |
| TGME49_268870 | tetratricopeptide repeat-containing protein | 114,008.90 | 5 | 6 | 8 | 6.50% |
| TGME49_309730 | thioredoxin reductase | 70,780.50 | 6 | 7 | 13 | 11.00% |
| TGME49_209950 | thioredoxin, putative | 47,700.60 | 9 | 11 | 14 | 23.60% |
| TGME49_293870 | thioredoxin, putative | 12,035.50 | 6 | 13 | 29 | 53.80% |
| TGME49_214080 | toxofilin | 26,789.10 | 6 | 10 | 36 | 30.90% |
| TGME49_229360 | transaldolase | 61,449.10 | 4 | 4 | 7 | 8.73% |
| TGME49_318310 | transketolase | 76,906.90 | 6 | 7 | 9 | 9.16% |
| TGME49_225930 | triose-phosphate isomerase TPI-I | 27,889.70 | 4 | 6 | 10 | 21.70% |
| TGME49_233500 | triose-phosphate isomerase TPI-II | 39,559.40 | 2 | 2 | 3 | 9.07% |
| TGME49_256970 | vacuolar ATP synthase subunit A, putative | 68,122.70 | 9 | 12 | 19 | 20.50% |
| TGME49_305290 | vacuolar atp synthase subunit e, putative | 39,083.80 | 3 | 3 | 5 | 9.86% |
| TGME49_253290 | valyl-tRNA synthetase | 125,051.80 | 2 | 2 | 3 | 2.62% |
| TGME49_244840 | zinc knuckle domain-containing protein | 24,157.50 | 2 | 2 | 3 | 12.30% |

|  |  |  |  |  |  |  |
| --- | --- | --- | --- | --- | --- | --- |
|  | **Common proteomics contaminates?** |  |  |  |  |  |
| TGME49_211290 | rhoptry protein ROP15 | 36,589.00 | 13 | 19 | 84 | 43.50% |
| TGME49_216750 | Paf1/RNA polymerase II complex  component LEO1 | 42,580.40 | 11 | 21 | 65 | 34.70% |
| TGME49_244210 | RNA polymerase-associated protein RTF1 | 84,700.20 | 13 | 18 | 74 | 18.60% |
| TGME49_244650 | eukaryotic initiation factor-5, putative | 46,804.90 | 7 | 13 | 54 | 18.80% |
| TGME49_249680 | RNA polymerase II associated Paf1 complex  component PAF1 | 61,780.70 | 10 | 17 | 74 | 19.60% |
| TGME49_300260 | threonyl-tRNA synthetase family protein | 95,900.80 | 10 | 10 | 26 | 10.30% |
| TGME49_288360 | tryptophanyl-tRNA synthetase (TrpRS2) | 77,060.60 | 10 | 11 | 25 | 13.60% |
| TGME49_306210 | RNA polymerase II assessory factor CDC73 | 77,627.10 | 21 | 28 | 113 | 33.90% |
| TGME49_312260 | hypothetical protein | 97,343.80 | 22 | 37 | 127 | 25.50% |
| TGME49_318230 | phosphoglycerate kinase PGKI | 44,676.90 | 11 | 17 | 41 | 34.80% |
| TGME49_314860 | zinc knuckle domain-containing protein | 76,149.60 | 5 | 7 | 12 | 12.00% |
| TGME49_308890 | transcription elongation factor SPT6 | 349,888.00 | 25 | 30 | 44 | 12.70% |
